# Broadly neutralizing antibodies target the coronavirus fusion peptide

**DOI:** 10.1126/science.abq3773

**Published:** 2022-07-12

**Authors:** Cherrelle Dacon, Courtney Tucker, Linghang Peng, Chang-Chun D. Lee, Ting-Hui Lin, Meng Yuan, Yu Cong, Lingshu Wang, Lauren Purser, Jazmean K. Williams, Chul-Woo Pyo, Ivan Kosik, Zhe Hu, Ming Zhao, Divya Mohan, Andrew J. R. Cooper, Mary Peterson, Jeff Skinner, Saurabh Dixit, Erin Kollins, Louis Huzella, Donna Perry, Russell Byrum, Sanae Lembirik, David Drawbaugh, Brett Eaton, Yi Zhang, Eun Sung Yang, Man Chen, Kwanyee Leung, Rona S. Weinberg, Amarendra Pegu, Daniel E. Geraghty, Edgar Davidson, Iyadh Douagi, Susan Moir, Jonathan W. Yewdell, Connie Schmaljohn, Peter D. Crompton, Michael R. Holbrook, David Nemazee, John R. Mascola, Ian A. Wilson, Joshua Tan

**Affiliations:** ^1^ Antibody Biology Unit, Laboratory of Immunogenetics, National Institute of Allergy and Infectious Diseases, National Institutes of Health, Rockville, MD 20852, USA.; ^2^ Department of Immunology and Microbiology, The Scripps Research Institute, La Jolla, CA 92037, USA.; ^3^ Department of Integrative Structural and Computational Biology, Scripps Research Institute, La Jolla, CA 92037, USA.; ^4^ Integrated Research Facility, Division of Clinical Research, National Institute of Allergy and Infectious Diseases, National Institutes of Health, Frederick, MD 21702, USA.; ^5^ Vaccine Research Center, National Institute of Allergy and Infectious Diseases, National Institutes of Health, Bethesda, MD 20892, USA.; ^6^ Integral Molecular, Philadelphia, PA 19104, USA.; ^7^ Clinical Research Division, Fred Hutchinson Cancer Research Center, Seattle, WA 98109, USA.; ^8^ Cellular Biology Section, Laboratory of Viral Diseases, National Institute of Allergy and Infectious Diseases, National Institutes of Health, Bethesda, MD 20892, USA.; ^9^ Protein Chemistry Section, Research Technologies Branch, National Institute of Allergy and Infectious Diseases, National Institutes of Health; Rockville, MD 20852, USA.; ^10^ Malaria Infection Biology and Immunity Section, Laboratory of Immunogenetics, National Institute of Allergy and Infectious Diseases, National Institutes of Health, Rockville, MD 20852, USA.; ^11^ New York Blood Center, Lindsley F. Kimball Research Institute, New York, NY 10065, USA.; ^12^ Flow Cytometry Section, Research Technologies Branch, National Institute of Allergy and Infectious Diseases, National Institutes of Health, Bethesda, MD 20892, USA.; ^13^ B Cell Immunology Section, Laboratory of Immunoregulation, National Institute of Allergy and Infectious Diseases, National Institutes of Health, Bethesda, MD 20892, USA.; ^14^ The Skaggs Institute for Chemical Biology, The Scripps Research Institute, La Jolla, CA, 92037, USA.

## Abstract

The potential for future coronavirus outbreaks highlights the need to broadly target this group of pathogens. We use an epitope-agnostic approach to identify six monoclonal antibodies that bind to spike proteins from all seven human-infecting coronaviruses. All six antibodies target the conserved fusion peptide region adjacent to the S2' cleavage site. COV44-62 and COV44-79 broadly neutralize alpha and beta coronaviruses, including SARS-CoV-2 Omicron subvariants BA.2 and BA.4/5, albeit with lower potency than RBD-specific antibodies. In crystal structures of Fabs COV44-62 and COV44-79 with the SARS-CoV-2 fusion peptide, the fusion peptide epitope adopts a helical structure and includes the arginine at the S2' cleavage site. COV44-79 limited disease caused by SARS-CoV-2 in a Syrian hamster model. These findings highlight the fusion peptide as a candidate epitope for next-generation coronavirus vaccine development.

Coronaviruses consist of four genera of viruses that infect birds and mammals (*1*). Seven coronaviruses are known to cause human disease: the alphacoronaviruses HCoV-229E and HCoV-NL63, as well as the betacoronaviruses HCoV-OC43, HCoV-HKU1, SARS-CoV, MERS-CoV and SARS-CoV-2. While the first four coronaviruses generally cause mild disease, the latter three have caused serious outbreaks in recent years. In particular, the ongoing coronavirus disease 2019 (COVID-19) pandemic by SARS-CoV-2 has resulted in more than six million deaths since the first cases were identified in 2019 (*2*). The currently dominant SARS-CoV-2 Omicron BA.2, BA.2.12.1 and BA.4/BA.5 subvariants are at least partially resistant to most available vaccines and antibody therapeutics (*3*–*6*). Furthermore, two coronaviruses previously linked only to animal infection were recently detected in individuals with flu-like symptoms (*7*, *8*). These developments highlight the importance of targeting conserved and functionally essential sites on coronaviruses.

Coronavirus infection is a multi-step process that involves enzymatic cleavage and rearrangement of the surface spike protein (*9*). The SARS-CoV-2 spike contains two cleavage sites: a furin cleavage site at the boundary of the S1 and S2 subunits, and an S2' site that is conserved in all coronaviruses. The spike protein is thought to be cleaved at the S1/S2 site during virus assembly, leaving the S1 and S2 subunits non-covalently linked. During entry, the SARS-CoV-2 spike protein uses the receptor-binding domain (RBD) on the S1 subunit to engage angiotensin-converting enzyme-2 (ACE2) on target cells. Following receptor binding, the S1 subunit is shed and the S2' site is cleaved by the membrane enzyme transmembrane serine protease 2 (TMPRSS2) or endosomal cathepsins (*1*), leading to the insertion of the fusion peptide into the cell membrane and viral fusion.

Much of the protection provided by COVID-19 vaccines arises from neutralizing antibodies that target the RBD (*10*). Likewise, all currently available therapeutic monoclonal antibodies (mAbs) target this domain (*3*). However, spike elements that participate in the subsequent stages of infection involve the more complex S2 fusion machinery with many moving parts and these elements are more conserved than the RBD, which so far has been capable of retaining or even increasing binding to ACE2 despite a variety of mutations (*11*). Therefore, these sites are worth exploring as targets for novel COVID-19 vaccines and therapeutics that retain efficacy against new variants and protect against a wider range of coronaviruses. Progress in this direction has started with recent studies identifying several mAbs that target the conserved stem helix (*12*–*16*) and using unbiased approaches to screen for mAbs of interest (*17*–*20*). Here we undertake a large-scale survey of the binding landscape of broadly reactive mAbs against coronaviruses.

## Identification of broadly reactive mAbs from COVID-19 convalescent donors

To identify individuals likely to harbor B cells that produce broadly reactive mAbs, we used a multiplex bead-based assay to examine plasma samples of 142 donors from a previously described cohort of COVID-19 convalescent individuals (*20*). We assessed plasma immunoglobulin G (IgG) reactivity toward spike glycoproteins of the seven human coronaviruses: SARS-CoV-2 (Wuhan Hu-1), SARS-CoV-1, MERS-CoV, HCoV-HKU1, HCoV-OC43, HCoV-NL63 and HCoV-229E. Nineteen donors were selected for mAb isolation and characterization based on plasma IgG reactivity to the spike proteins of SARS-CoV-2 and at least two other betacoronaviruses (fig. S1A).

**Fig. 1.**
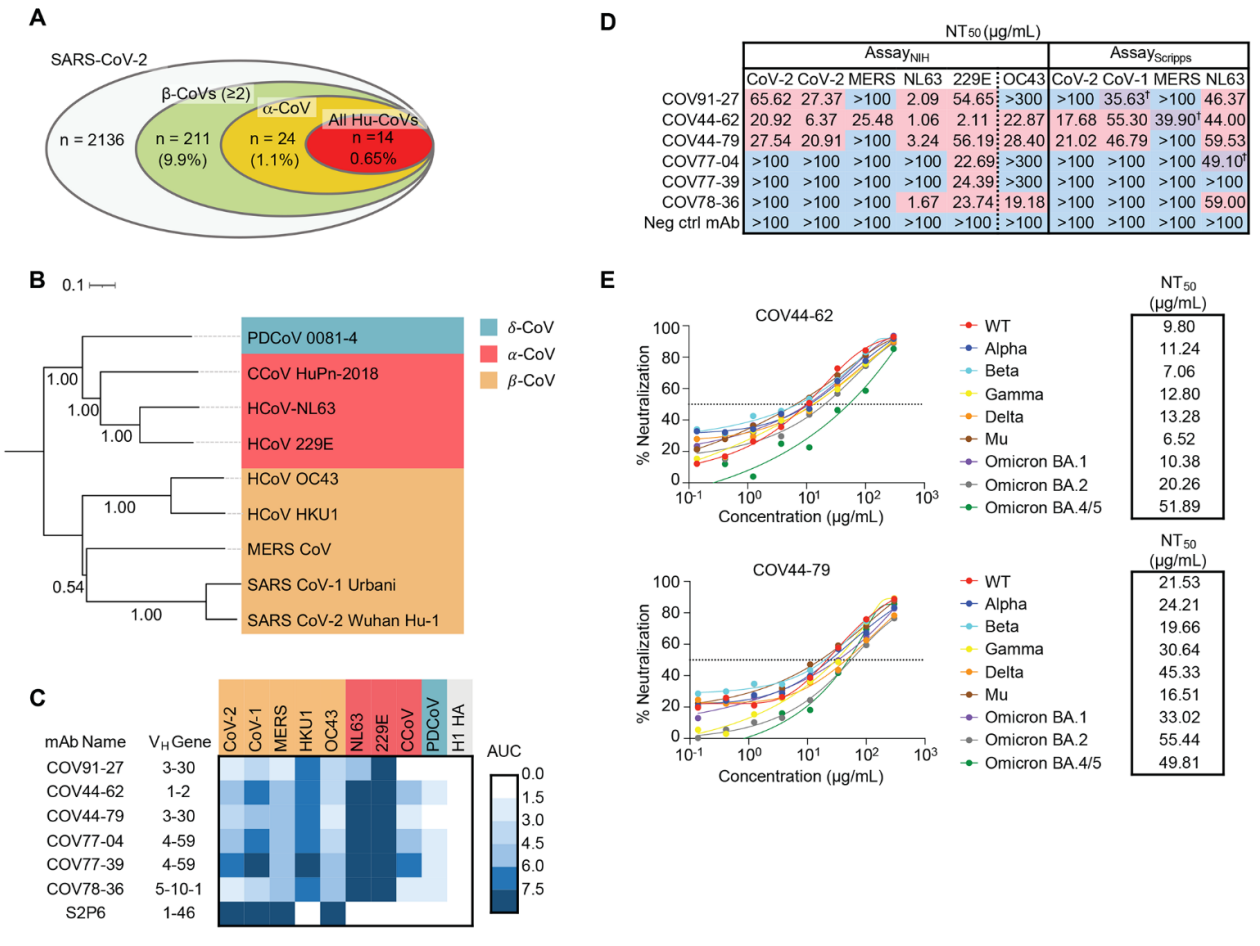
Broadly neutralizing antibodies target coronaviruses associated with human disease. (**A**) Analysis of the frequency of MBCs expressing broadly reactive antibodies from *n* = 19 donors. Values in parentheses represent the percentage of SARS-CoV-2 reactive supernatants that also bind the specified subsets of non-SARS coronavirus spikes. A total of 10,356 MBC culture supernatants (50-100 B cells/well) was screened. (**B**) Phylogenetic relationships across the coronavirus spike proteins targeted by the broadly reactive mAbs were inferred by the Neighbor-Joining method in MEGA11 using full-length amino-acid sequences of CoV spike proteins. Branch lengths are drawn to scale and bootstrap values from 500 samplings are shown on the branches. The scale bar represents the number of amino acid substitutions per site. (**C**) Heat map representing the binding of broadly reactive mAbs to spike proteins from coronaviruses across the alpha, beta and deltacoronavirus genera. H1 hemagglutinin was included as a negative control for mAb binding experiments and area under the curve (AUC) values for each antigen are shown after subtraction with values for the negative control antigen CD4. (**D**) Values represent antibody titer at 50% neutralization (NT_50_) against SARS-CoV-2 Wuhan Hu-1, SARS-CoV-1, MERS-CoV, HCoV-NL63 and HCoV-229E envelope-pseudotyped lentivirus, as well as authentic HCoV-OC43. For Assay_Scripps_, values are average of two experiments. For values with the † symbol, one NT_50_ was determinable and one was not (i.e., >100 μg/mL), and the determinable NT_50_ is shown. Negative controls mAbs were anti-CoV-2 RBD CV503 (for OC43 assay) (*20*), anti-influenza HA CR9114 (for Assay_NIH_ except OC43) (*41*) and anti-dengue DEN3 (for Assay_Scripps_) (*42*). NT50 values were calculated using the dose-response-inhibition model with 5-parameter Hill slope equation in GraphPad Prism 9. (**E**) Neutralization of SARS-CoV-2 variants of concern (pseudovirus) by COV44-62 and COV44-79.

We next interrogated human IgG^+^ memory B cells (MBCs) from the selected donors using a two-stage screen to prioritize isolating mAbs with the greatest possible breadth of reactivity. First, we screened supernatants from 673,671 stimulated IgG^+^ B cells for binding to the coronavirus spike panel used in the plasma screen. Supernatants from only 2% (*n* = 211) of the MBC culture wells met our criteria for broad reactivity by binding at least three betacoronavirus spike proteins (Fig. 1A). Next, we developed an optofluidics assay to isolate individual MBCs of interest using the Berkeley Lights Beacon system (fig. S1B). Candidate MBCs identified in the supernatant screen were sorted individually into nanoliter-volume pens and assessed in real-time for secretion of mAbs that bound to beads coated with a cocktail of MERS-CoV and HCoV-OC43 spikes, followed by beads coated with SARS-CoV-2 spike. Double positive MBCs were exported for single-cell reverse transcription-PCR (RT-PCR) and antibody production as recombinant IgG1. In total, we obtained 60 IgG mAbs with reactivity to at least three coronaviruses.

To fully interrogate their breadth, we tested the 60 mAbs for binding to spikes from the seven human coronaviruses. Only six mAbs, COV91-27, COV44-62, COV44-79, COV77-04, COV77-39 and COV78-36, bound to spike proteins from all seven coronaviruses (Fig. 1, B and C). Notably, four of the six also bound to spike from two new coronaviruses recently associated with human disease: Canine CoV HuPn-2018 (CCoV-HuPn-2018) and Porcine Deltacoronavirus 0081-4 (PDCoV-0081-4) (*7*, *8*) (Fig. 1C). The six broadly reactive mAbs were isolated from four different donors and were encoded by four different heavy chain variable (VH) genes (VH1-2, VH3-30, VH4-59, VH5-10-1) (table S1). Five mAbs were highly mutated, with VH nucleotide mutation frequencies ranging from 10 to 13% (fig. S1C). Given that these mAbs were isolated from COVID-19 convalescent individuals in New York approximately one month after the first outbreak in March 2020, these mutation levels suggest that the B cells were primed during an earlier seasonal coronavirus infection and possibly reactivated during SARS-CoV-2 infection.

## COV44-62 and COV44-79 broadly neutralize coronaviruses

We assessed the neutralizing potency of the six mAbs against SARS-CoV-2, SARS-CoV-1, MERS-CoV, HCoV-NL63 and HCoV-229E envelope pseudotyped viruses, as well as authentic HCoV-OC43. COV44-62 and COV44-79 showed the broadest functional reactivity, neutralizing the betacoronaviruses SARS-CoV-2, SARS-CoV-1 and HCoV-OC43, as well as the alphacoronavirus HCoV-NL63 and HCoV-229E (Fig. 1D and fig. S1D). Moreover, both mAbs neutralized SARS-CoV-2 variants of concern, including the Omicron BA.2 and BA.4/5 subvariants, as well as authentic SARS-CoV-2 (Fig. 1E and fig. S1E). COV44-62 also neutralized MERS-CoV, whereas no other mAbs neutralized this virus within the concentrations tested.

## Broadly reactive mAbs target the coronavirus fusion peptide

**Fig. 2.**
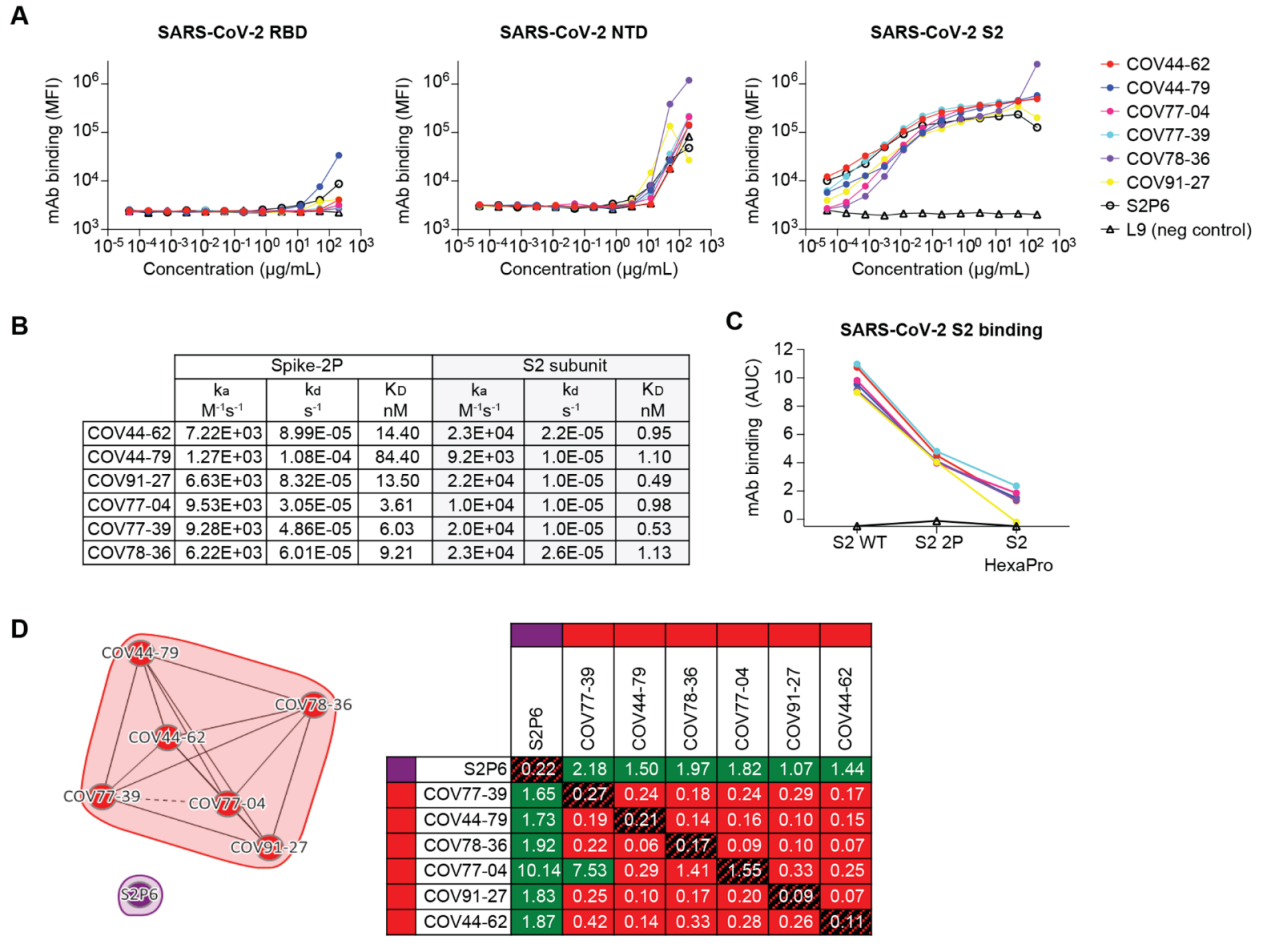
Broadly reactive mAbs target the same region within the SARS-CoV-2 S2 subunit. (**A**) Titration curves for mAb binding to selected regions within the SARS-CoV-2 spike protein: the receptor binding domain (RBD), N-terminal domain (NTD) and the S2 subunit. Interconnected data points are shown without curve fitting. L9 is a malaria-specific mAb used as a negative control (*43*). (**B**) On-rates, off-rates and dissociation constants of the six fusion peptide Fabs for binding to SARS-CoV-2 pre-fusion stabilized spike (2P) with an unmodified furin cleavage site and the non-stabilized S2 subunit. (**C**) Fusion peptide mAb binding (AUC) to wild-type SARS-CoV-2 S2 subunit and S2 subunit constructs modified with two (2P) or six (HexaPro) stabilizing proline mutations. (**D**) Epitope binning of broadly reactive antibodies versus the S2 stem-helix targeting mAb S2P6. All included antibodies were tested as both ligands and analytes. Solid lines indicate two-way competition while hashed lines indicate one-way competition. Red boxes indicate competing antibody pairs, green boxes indicate non-competing antibody pairs and hashed filling indicates self-competition.

To determine the domain of SARS-CoV-2 spike that was targeted by the six broadly reactive mAbs, we assessed mAb binding to the SARS-CoV-2 S2 subunit, as well as the RBD and N-terminal domain (NTD) of the S1 subunit. All six mAbs bound only to the S2 subunit (Fig. 2A). VJ germline-reverted versions of the broadly neutralizing mAbs COV44-62 and COV44-79 showed weaker binding to S2 than the mutated versions (fig. S2A), suggesting that somatic mutations were important for improving binding to the target site. A surface plasmon resonance (SPR) kinetics assay determined the binding affinity of antigen-binding fragments (Fabs) derived from these mAbs for pre-fusion stabilized whole SARS-CoV-2 spike (2P, with an intact S1/S2 cleavage site) and the unmodified S2 subunit. The Fabs bound with low-to-moderate nanomolar affinity to both proteins, but their affinity for the S2 subunit was 3- to 76-fold higher than their affinity for whole spike (Fig. 2B and fig. S2A). There were no substantial differences between the six Fabs in their affinity for the S2 subunit. The mAbs showed reduced binding to a form of S2 that had been stabilized with two proline mutations (S2-2P) and bound more poorly still to a further stabilized version with six proline mutations (S2-HexaPro) (Fig. 2C). SPR-based competition showed that the six mAbs competed for the same binding site on the S2 subunit (Fig. 2D), but none competed with S2P6, a control mAb targeting the stem helix region (*12*), indicating their specificity for a distinct site on S2.

**Fig. 3.**
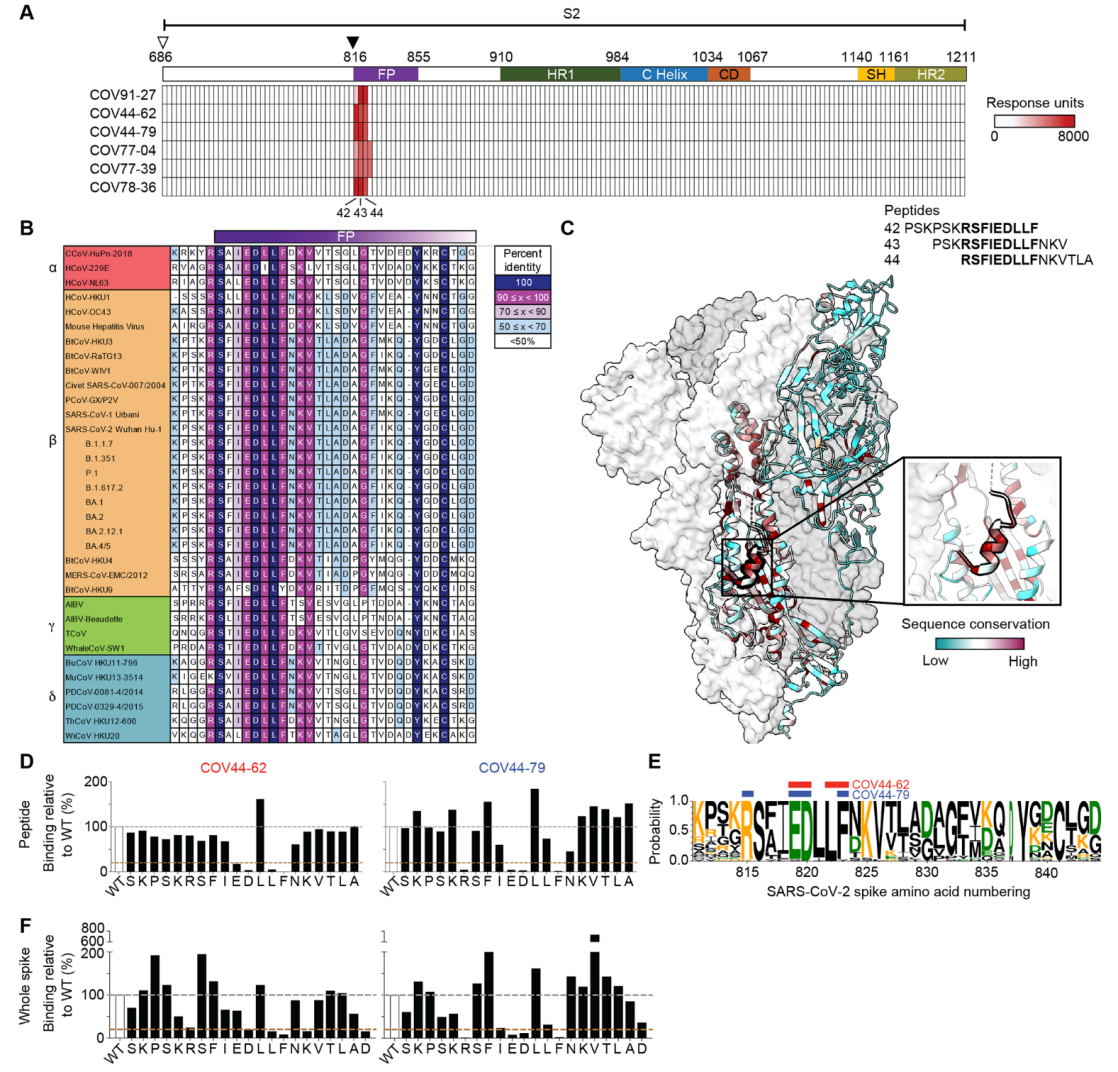
Broadly neutralizing antibodies target the conserved fusion peptide. (**A**) Heat map of SARS-CoV-2 S2 peptide array. Binding responses were assessed by SPR using a 15-mer peptide array with 12 aa overlay covering the entire S2 subunit. Each column within the map represents a single peptide. The white triangle shows the S1/S2 cleavage site and the black triangle indicates the S2' cleavage site. FP, fusion peptide; HR1, heptad repeat 1; C helix, central helix; CD, connector domain; SH, stem helix; HR2, heptad repeat 2. (**B**) Sequence alignment of the fusion peptide from 34 viral isolates representing a diverse group of coronaviruses across four genera. Performed using MAFFT v7.450 using a BLOSUM62 scoring matrix and the L-INS-I algorithm. (**C**) Sequence conservation of pre-fusion SARS-CoV-2 spike protein (PDB 6VSB) with the fusion peptide (aa 816-843) highlighted in a black outline. Inset shows a magnified view of this region. (**D**) Alanine scan evaluating the binding of COV44-62 and COV44-79 to the SARS-CoV2 fusion peptide. Responses were normalized to the wild-type sequence. A cut-off of 20% (brown hashed line) was used to identify residues that were critical for binding. (**E**) Sequence logo plot of diversity within the fusion peptide region of coronaviruses from 34 isolates, built using WebLogo 3. Height is proportional to the probability of an amino acid at a given position and amino-acid residues are colored by charge. Narrow stacks (amino acids) indicate deletions or gaps in the sequences. Numbering is based on the SARS-CoV-2 Wuhan-Hu-1 sequence. The key residues in the epitope footprints of mAbs COV44-62 (red) and COV44-79 (blue), based on peptide alanine scanning, are highlighted above the logo plot. (**F**) Amino acids critical for the binding of COV44-62 and COV44-79 identified by shotgun alanine mutagenesis of S2 residues on whole spike protein. Only fusion peptide residues are shown here. Key residues were identified based on a <20% signal relative to wild-type spike (brown hashed line), with no corresponding loss of signal for a control mAb, which targets the spike protein but does not bind to this site (see fig. S3C).

To further interrogate the specificity of these mAbs, we performed SPR-based peptide mapping using an array of 15-mer overlapping peptides that spanned the entire SARS-CoV-2 S2 subunit (Ser686 to Lys1211, Accession #YP_009724390.1). All six mAbs bound to peptides 42-44, which share the _815_RSFIEDLLF_823_ motif (Fig. 3A). This motif is located within the SARS-CoV-2 fusion peptide region, directly C-terminal to the S2' cleavage site. To determine the diversity of this region across coronaviruses, we selected 34 viral isolates representing each of the coronavirus genera – *alpha*, *beta*, *gamma*, and *delta* (Fig. 3B). Each amino-acid position in the _815_RSFIEDLLF_823_ motif was conserved in >90% of all viruses selected except F_817_, which was conserved in <50% of isolates examined (Fig. 3, B and C). The fusion peptide appears partially surface-exposed in a range of coronavirus spike proteins, including SARS-CoV-2, SARS-CoV, MERS-CoV and MHV (*21*, *22*) (Fig. 3C and fig. S3A). However, antibody access to this site may be partially occluded by the S1 subunit on an adjacent protomer, consistent with their stronger binding to the S2 subunit relative to SARS-CoV-2 spike (Fig. 2B and fig. S3B).

That these mAbs target the fusion peptide is consistent with their reduced binding to the HexaPro S2 construct (Fig. 2C), which includes a non-conservative F817P mutation at this site (*23*). To identify critical amino acids for mAb binding, we performed an alanine scan on a peptide encompassing residues 810 to 830 and focused on residues targeted by the broadly neutralizing mAbs COV44-62 and COV44-79 (Fig. 3D). Four amino acids, E819, D820, L822 and F823, were important for binding of COV44-62, where mutation of the F823 residue abolished binding. Similarly, critical residues for the binding of COV44-79 were E819, D820 and F823, but also included R815 at the S2' cleavage site (Fig. 3D). All five residues identified as important for COV44-62 or COV44-79 binding are among the most conserved residues in the coronavirus spike protein. D820 and L822 are completely conserved, while R815, E819 and F823 are conserved in 34 out of 35 coronaviruses (Fig. 3, B and E). Amino-acid mutations at the peptide level may have different effects from mutations in the intact spike protein, where modified interactions with surrounding residues may also affect antibody binding. Therefore, we screened the six mAbs using a shotgun alanine mutagenesis approach, whereby every amino acid in the S2 subunit of intact spike was individually mutated to generate a panel of spike mutants (Fig. 3F and fig. S3, C and D). In general, this assay identified a greater number of residues as important for mAb binding, including some with a more intermediate phenotype. For COV44-62, D820, L822 and F823 were again crucial for binding, while K825 and D830, as well as R815, were also identified as important (Fig. 3F). For COV44-79, the results closely matched the peptide alanine scan, with the same four amino acids, R815, E819, D820 and F823, identified as the most critical. When the six mAbs were analyzed as a group, we found that only the four broadest neutralizing mAbs were negatively affected by the R815A mutation (fig. S4, A and B), suggesting that binding to the S2' cleavage site may be a distinguishing property of broadly neutralizing mAbs against this site.

## Crystal structures of anti-fusion peptide antibodies

**Fig. 4.**
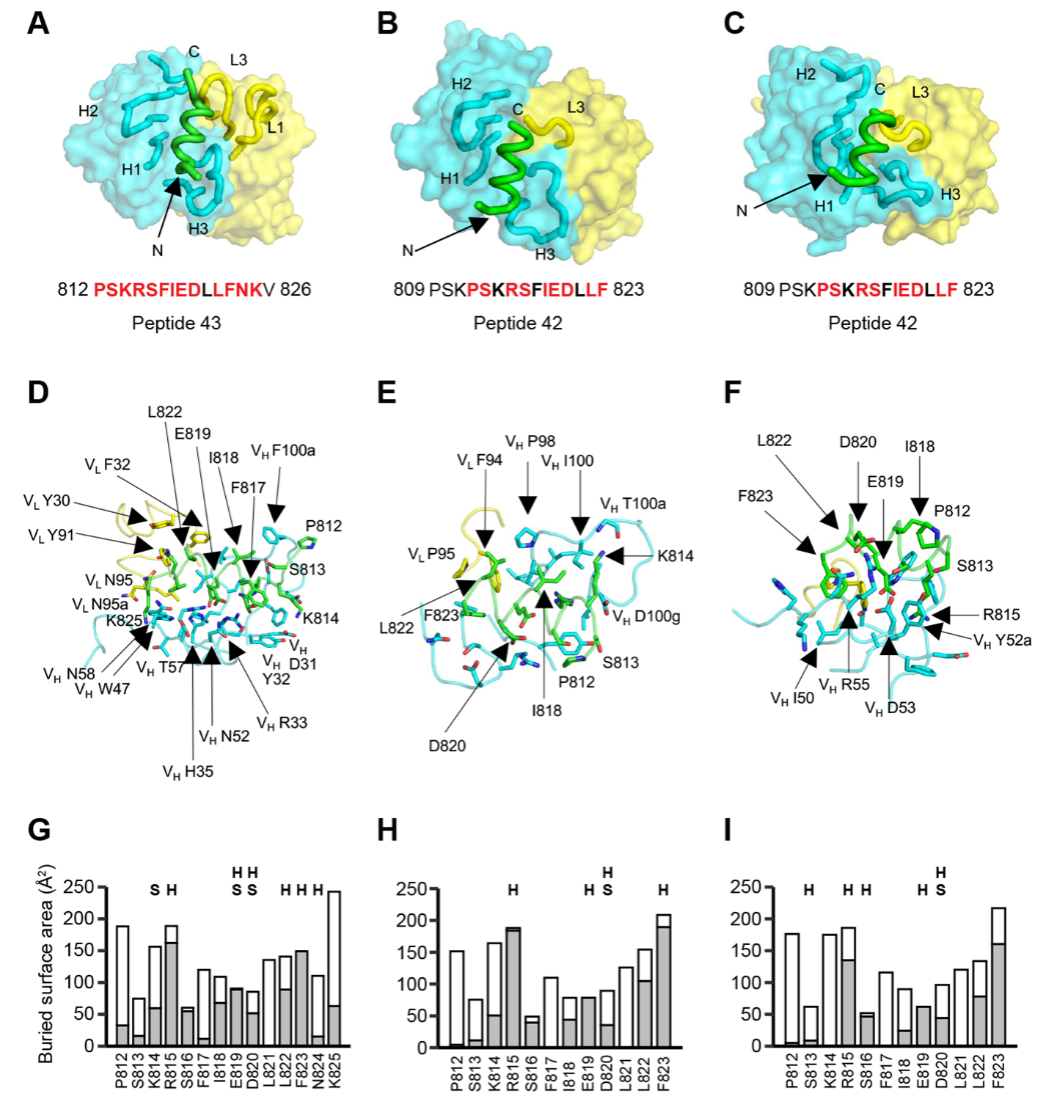
Crystal structures of COV44-62, COV44-79, and COV91-27 in complex with SARS-CoV-2 fusion peptide. (**A **to** C**) The overall interactions of (A) COV44-62, (B) COV44-79, and (C) COV91-27 with the fusion peptide. Fabs are shown in a molecular surface and the CDRs and peptides are represented as tubes. Cyan and yellow represent the heavy and light chains of the Fabs. Peptides are shown in green. H1, H2, H3, L1, and L3 denotes CDRs in the heavy (H) and light (L) chains. The resolution of the crystal structures are 1.46 Å, 2.8 Å, and 2.3 Å for the COV44-62, COV44-79, and COV91-27 complexes. Peptide residues observed in the crystal structure are in bold and residues involved in interaction with antibody (buried surface area >0 Å^2^) are in red. (**D **to** F**) Details of the interactions between (D) COV44-62, (E) COV44-79, and (F) COV91-27 with the fusion peptide. V_H_ and V_L_ indicate the variable domains of the heavy (H) and light (L) chains. Kabat numbering was used for the Fabs and numbering in the native spike protein for the fusion peptide. The colors for the heavy chain, light chain, and fusion peptide are as in (A). (**G **to** I**) Buried surface area (in gray) and accessible surface area (in white) of each residue of the fusion peptide in complex with antibody are shown in the stacked bar chart. Residues that form polar interactions with COV44-62, COV44-79, and COV91-27 are denoted with “H” if they form a hydrogen-bond or “S” for a salt bridge on top of the corresponding bar. Buried and accessible surface areas were calculated with PISA (*44*).

To elucidate the molecular characteristics of anti-fusion peptide antibodies that neutralize SARS-CoV-2, the Fabs of the three broadest neutralizing mAbs COV44-62, COV44-79, and COV91-27 were complexed with 15-mer peptides containing the fusion peptide sequence (Fig. 4). Crystal structures were determined to 1.46 Å, 2.8 Å and 2.3 Å resolution, respectively (Fig. 4, fig. S5 and table S2). Fourteen of the 15 peptide residues were visible in the electron density map for COV44-62 (fig. S5A), 13 of which have a buried surface area (BSA) >0 Å^2^ in complex with antibody. For COV44-79, 12 of the 15 peptide residues were visible (fig. S5A), 10 of which have a buried surface >0 Å^2^. Similarly, in COV91-27, 12 peptide residues had interpretable density (Fig. 4C and fig. S5A), with nine exhibiting a buried surface >0 Å^2^. The fusion peptide forms a helix as in the prefusion state of the SARS-CoV-2 spike (fig. S5B). All three complementarity-determining regions (CDRs) of the heavy chain (HC) of all three Fabs are involved in peptide recognition whereas CDR1 and CDR3 of the light chain (LC) of COV44-62 and only LCDR3 of COV44-79 and COV91-27 contact the peptide (Fig. 4, A to C). The BSA on each Fab is dominated by the heavy chain and is 791 Å^2^ for COV44-62 (627 Å^2^ by HC and 164 Å^2^ by LC), 573 Å^2^ for COV44-79 (505 Å^2^ by HC and 129 Å^2^ by LC), and 573 Å^2^ for COV91-27 (447 Å^2^ by HC and 126 Å^2^ by LC). The fusion peptide makes side-chain and backbone H-bonds and salt bridges with COV44-62 mainly through K814, R815, E819, D820, L822, F823 and N824, and hydrophobic interactions through I818, L822, and F823 (Fig. 4D). These residues include the key residues, E819, D820, L822 and F823 identified by site-directed mutagenesis (Fig. 3D). The fusion peptide did not form as many interactions with COV44-79 and COV91-27 (Fig. 4, E and F). However, R815, E819, and D820 contributed H-bonds and salt bridges, and I818, L822, and F823 made hydrophobic interactions. In all three antibodies, R815, S816, I818, E819, D820, L822, and F823 contributed the most buried surface area to the interaction (Fig. 4, G to I). There was partial overlap between the residues of COV44-62 and COV44-79 that interacted with the fusion peptide and those that were mutated from germline (fig. S6), consistent with the reduction in binding of germline-reverted versions of these mAbs (fig. S2A). The structural results are consistent with the mutagenesis data with peptide and spike protein that identify the key binding residues (Fig. 3, D to F, and fig. S3D). Importantly, the arginine at the S2' cleavage site is involved in recognition by these anti-fusion peptide antibodies. Although the antibodies all interact with one face of the fusion peptide helical structure (fig. S5D), their approach angles to the peptide differ. Superimposition of the fusion peptide structures onto an intact SARS-CoV-2 spike trimer structure in the pre-fusion state showed a potential clash with the S protein, suggesting that a conformational change or conformational dynamics around the fusion peptide is required to accommodate antibody targeting (fig. S5C). Notwithstanding, these antibodies have neutralization activity against SARS-CoV-2 and therefore are able to interact with the fusion peptide on the virus (Fig. 1D).

## Response to the fusion peptide following vaccination and infection

We compared the binding of polyclonal IgG from mRNA-1273-vaccinated donors (fig. S7A), COVID-19 convalescent individuals, and COVID-19-naïve individuals to the SARS-CoV-2 fusion peptide (peptide 43) (Fig. 3A and fig. S7B). All COVID-19-naïve donors showed minimal binding to the peptide, indicating a minor contribution by previous seasonal coronavirus infections to circulating fusion peptide-specific IgG. While there was an increased response in several vaccinees after the 2nd vaccine dose (*P* = 0.025), this was not enhanced by the administration of a booster. The COVID-19 convalescent donors did not have significantly higher responses than vaccinated donors (*P* = 0.864 versus post-2nd dose). However, several convalescent donors had the highest responses in all three cohorts, suggesting that SARS-CoV-2 infection triggers a strong fusion peptide-specific antibody response in some individuals.

## COV44-62 and COV44-79 inhibit membrane fusion

**Fig. 5.**
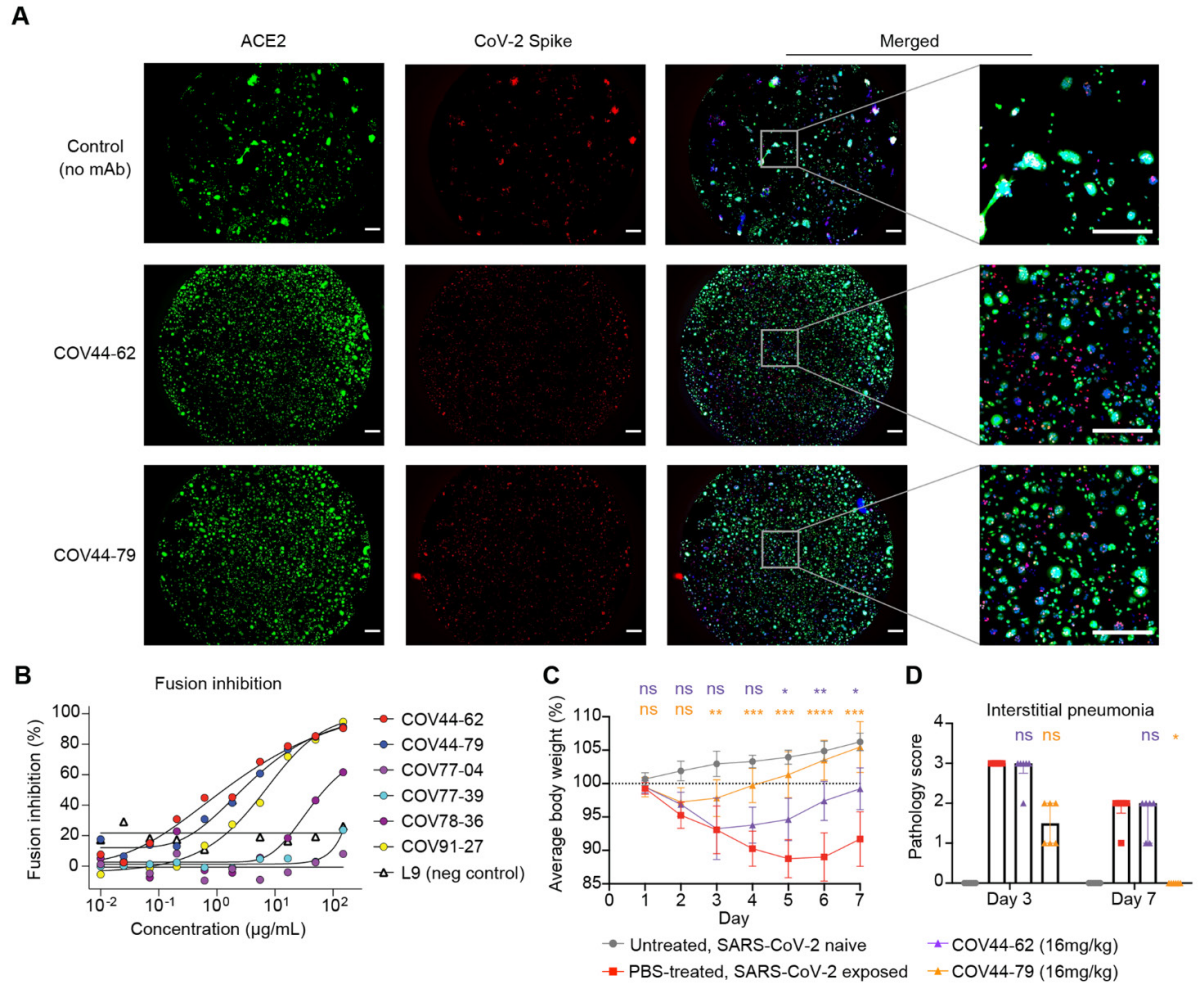
COV44-62 and COV44-79 inhibit SARS-CoV-2 spike-mediated fusion and COV44-79 limits disease in a Syrian hamster model. (**A**) Images of fusion between HeLa cells stably expressing SARS-CoV-2 spike (RFP) and HeLa cells stably expressing the ACE2 receptor (GFP) after counter-staining with Hoechst (blue). Cells were co-cultured in the presence of COV44-62, COV44-79 or without a mAb (control). Scale bar, 500 μm. (**B**) Fusion inhibition of six fusion peptide-specific mAbs in a quantitative assay. (**C**) Weight change for SARS-CoV-2 naïve animals versus virus-exposed animals that were mock-treated or treated with 16 mg/kg of mAb. Statistical significance for average body weight was analyzed across the 7-day time-course using a mixed-effects repeated measures model with Dunnett's post-test multiple comparison (*n* = 12 animals from Day 0-3 and *n* = 6 animals from Day 4-7). Error bars show mean ± SD. (**D**) Pathology scores for SARS-CoV-2 naïve animals versus virus-exposed animals that were mock-treated or treated with 16 mg/kg of mAb. Scores for interstitial pneumonia pathology (Days 3 and 7) based on gross pathology observations were statistically analyzed by a Kruskal-Wallis test with Dunn’s post-test multiple comparison (*n* = 6-12 animals per condition), between the mAb-treated and mock-treated groups on each day. *P < 0.05, **P < 0.01, ***P < 0.001, ****P < 0.0001 and ns, not significant. Bars show median + interquartile range.

Spike-mediated cell fusion relies on insertion of the fusion peptide into the target cell membrane and thus might be inhibited by antibody binding to the fusion peptide. Consistent with this, COV44-62 and COV44-79 inhibited the fusion of cells expressing SARS-CoV-2 spike and cells expressing the ACE2 receptor in an imaging-based assay (Fig. 5A). We further tested the six fusion peptide-specific mAbs in a more quantitative assay wherein fusion would trigger the release of an enzyme that cleaves a chromogenic substrate. Only the mAbs that neutralized SARS-CoV-2 were able to strongly inhibit fusion (Fig. 5B).

## COV44-79 limits disease in the Syrian hamster model

We evaluated the in vivo efficacy of COV44-62 and COV44-79 against SARS-CoV-2 infection in the Syrian hamster model, a well-established model recapitulating features of moderate to severe COVID-19 in humans (*24*–*26*). We converted the Fc regions of the two mAbs to hamster IgG2 to allow optimal Fc function. The mAbs were administered intraperitonially at 16 mg/kg, followed by intranasal administration of 5 log10 PFU of SARS-CoV-2 WA01 24 hours later (Fig. 5, C and D, and fig. S8). Hamsters treated with COV44-79, and to a lesser extent COV44-62, had a smaller decrease in body weight and recovered more quickly than untreated hamsters (*P* < 0.01 from days 3-7 for COV44-79 and *P* < 0.05 from days 5-7 for COV44-62) (Fig. 5C). Similar results were observed in a second experiment where COV44-79 was tested in comparison to a hamster IgG2 isotype control (fig. S7B). Furthermore, semiquantitative scoring revealed that hamsters treated with COV44-79 had less interstitial pneumonia than untreated hamsters on day 7 (*P* < 0.05) (Fig. 5D). COV44-79 was also able to slightly reduce lung viral titers relative to control hamsters based on sub-genomic RNA quantification and plaque assay analysis (fig. S8C).

## Discussion

The broad conservation and functional importance of the fusion peptide highlight the potential of this site as a candidate for coronavirus vaccine development. These findings have parallels to work done on HIV-1 gp120, where the surface-exposed fusion peptide was identified as a target of neutralizing mAbs (*27*). This discovery led to the investigation of the HIV-1 fusion peptide as a candidate immunogen to elicit broadly neutralizing antibodies in animals (*28*); subsequent animal studies have increased the potency and breadth of the fusion peptide-targeting antibody response (*29*). Despite these potential advantages, the coronavirus fusion peptide has not been a major focus for development of therapeutic mAbs and COVID-19 vaccines. The main drawback of the mAbs described here is their comparatively low in vitro neutralization potency. These mAbs fit into a wider trend of a trade-off between potency and breadth: highly potent mAbs targeting the RBD are restricted to sarbecoviruses and most do not neutralize all variants of SARS-CoV-2 (*3*, *5*, *6*, *20*, *30*), while mAbs targeting the stem helix (*12*–*16*) and those identified here have greater breadth but are less potent. However, as previously reported for at least one anti-stem helix mAb (*15*), COV44-79 performed better in the hamster model than expected, suggesting that it may function in a way that is not captured effectively in the neutralization assay. For instance, Fc effector functions may enhance activity, as observed with anti-SARS-CoV-2 antibodies with other specificities (*31*–*33*). Moreover, there is substantial scope for improvement for mAbs with this specificity and subsequent studies may discover mAbs with higher potency, as with the HIV-1 fusion peptide (*34*). Techniques to improve antibody affinity and potency could also be useful (*35*, *36*).

Additionally, vaccination with fusion peptide constructs may trigger a polyclonal response of greater magnitude and potency. We found that three doses of the mRNA-1273 vaccine did not produce strong antibody responses to the fusion peptide although several COVID-19 convalescent individuals was strong antibody responses to this site. This observation is consistent with greater exposure of the S2 subunit to B cells during natural infection due to S1 uncoupling, which likely occurs less frequently with pre-fusion stabilized spike protein. Furthermore, depletion of fusion peptide-specific antibodies from the serum of COVID-19 convalescent patients resulted in a 20% reduction in SARS-CoV-2 neutralization (*37*), and reactivity to the fusion peptide correlated with neutralization titer (*38*), indicating that polyclonal antibodies to the fusion peptide can play an appreciable role. Thus, our findings are consistent with previous studies highlighting the potential utility of the fusion peptide as a target epitope (*37*–*40*) and the fusion peptide-targeted mAbs provide additional tools to combat COVID-19 and enhance pandemic preparedness.

## Supplementary Materials

### This PDF file includes:

Materials and Methods Figs. S1 to S8 Tables S1 and S2 References (*45*–*69*)

### Other Supplementary Material for this manuscript includes the following:

MDAR Reproducibility Checklist
