# Supplementary material for: Broadly neutralizing antibodies target the coronavirus fusion peptide
Source: Science. 2022 Jul 12:eabq3773. doi: 10.1126/science.abq3773 (PMC9348754; doi:10.1126/science.abq3773)
Supplement: Supplementary file 1 — Materials and Methods Figs. S1 to S8 Tables S1 and S2 References ( 45 – 69 ) [file science.abq3773_sm.pdf]

Science
AAAS

Supplementary Materials for

Broadly neutralizing antibodies target the coronavirus fusion peptide

Cherrelle Dacon *et al.*

Corresponding author: Joshua Tan, tanj4@nih.gov

DOI: 10.1126/science.abq3773

The PDF file includes:

Materials and Methods
Figs. S1 to S8
Tables S1 and S2
References

Other Supplementary Material for this manuscript includes the following:

MDAR Reproducibility Checklist

Materials and Methods

Study participants

For coronavirus disease 2019 (COVID-19) convalescent participants, whole blood and plasma samples were obtained from a previously described cohort (*20*). All participants in this study met the inclusion criteria of ≥ 18 years of age, reverse transcription-PCR (RT-PCR) confirmation of SARS-CoV-2 infection while symptomatic and at least 2 weeks elapsed between their last report of COVID-19 symptoms and the time of blood collection. All participants in the convalescent cohort provided informed consent for their blood products to be used for research purposes by signing the standard New York Blood Center (NYBC) blood donor consent form. Plasma samples from all 142 participants were examined in the initial screen and 19 participants were selected for inclusion in the current study based on plasma immunoglobulin G (IgG) reactivity. For participants who received the SARS-CoV-2 mRNA-1273 vaccine (Moderna), whole blood, plasma and serum samples were obtained at the NIH Clinical Research Center in Bethesda, MD under protocols approved by the NIH Institutional Review Board, ClinicalTrials.gov identifiers: NCT00001281 and NCT05078905. All 16 participants in the vaccine study met the inclusion criteria of ≥ 18 years of age, no known history of SARS-CoV-2 infection (based on nucleocapsid antibody responses) and had not previously received a dose of a COVID-19 vaccine at the time of enrollment. Blood samples were serially drawn from all 16 vaccinees: prior to administration of the first mRNA-1273 dose (baseline), 30 days post-administration of the 2nd dose, prior to administration of the 3rd dose (pre-booster baseline) and 30 days after the 3rd dose. For 3 participants, an additional blood draw was collected 30 days post-documented SARS-CoV-2 infection. All participants in the mRNA-1273 cohort provided written informed consent to have their blood products used for research purposes. No randomization or blinding was applied to the analysis of participants' plasma, serum or peripheral blood mononuclear cell (PBMC) samples, but all samples were anonymized before being used in this study.

Coronavirus spike proteins

Spike proteins of HCoV-NL63 spike (Sino Biological, 40604-V08B-B) HCoV-229E spike (Sino Biological 40605-V08B-B) and HCoV-HKU1 (Sino Biological, 40606-V08B) were commercially acquired. The HCoV-OC43 spike and MERS-CoV spike were synthesized as previously described

(*45*) and kindly gifted by Andrew Ward. Briefly, both ectodomain constructs contain a C-terminal T4 fibritin trimerization domain, an HRV3C cleavage site, an 8× His-Tag, and a Twin-strep-tag for purification. For protein expression, Freestyle™ 293-F cells (Thermo Scientific, R79007) were transfected with the spike plasmid of interest, and cultures were harvested at 6-days post-transfection. The spike proteins were purified from the supernatants on cOmplete™ His-Tag Purification resin before further purification with Superose 6 increase (S6i) 10/300 column (GE Healthcare Biosciences). Pre-fusion stabilized constructs for CCoV HuPn-2018 (Accession # QVL91811, aa1-1384 with E1140P and E1141P mutations) and PdCoV0081-4 (Accession # MW685622, aa1-1092 with E854P and V855P mutations) were synthesized and cloned into pCDNA3.1- vectors (Genscript) with the following C-terminal modifications: T4 fibritin trimerization motif, HRV3C protease cleavage site, poly-GS linker, Avi-tag, and 8× His tag. Freestyle™ 293-F cells (Thermo Scientific, R79007) were transfected with 3:1 ratio of spike plasmid and PEIMax, pH 7 (Polysciences, 24765). At day 3, cells were supplemented with soy hydrolase and glucose. 5-7 days post-transfection, the supernatant was harvested, clarified, and purified using a His-Trap Excel Column (Cytiva).

SARS-CoV-2 N-terminal domain (NTD), receptor-binding domain (RBD), and spike, as well as SARS-CoV RBD and spike, were expressed and purified as previous described (*20, 46, 47*). Briefly, the SARS-CoV-2 NTD and RBD were cloned into an in-house pFastBac vector. The vector was fused with a gp67 signal peptide and an His_6 tag flanking the N- and C-terminus of the NTD and RBD. The recombinant bacmids were obtained from Bac-to-Bac system (Life Technologies). Baculoviruses were produced by transfection of bacmid DNA into Sf9 cells and used to infect High Five cells (Life Technologies, B85502) at high (5 to 10) multiplicity of infection (MOI). The supernatant of the infected High Five cells was harvested around 72 hours post-infection at 28°C with shaking at 110 rpm.

The SARS-CoV-2 S HexaPro plasmid was a generous gift from Jason McLellan (Addgene plasmid # 154754) (*23*). The spike S2 domain (699 to 1207 with F817P, A892P, A899P, A942P, K986P, V987P) was constructed into a phCMV3 vector which contained an N-terminal secreting signal peptide, and C-terminal thrombin cleavage site and His_6 tag. Proteins were purified by Ni Sepharose excel resin (Cytiva) followed by size exclusion chromatography (SEC) in 20 mM Tris buffer with 150 mM NaCl, pH 7.4.

Expression and purification of the recombinant hemagglutinin (HA) were conducted as previously described (*41, 48*). The ectodomain (11-329 of HA1 and 1-174 to HA2, in H3 numbering) of A/Solomon Islands/03/2006 (H1N1) HA was fused to an N-terminal gp67 signal peptide and a C-terminal BirA biotinylation site, thrombin cleavage site, T4 trimerization domain and 6xHis-tag of a customized pFastBac vector. The Bac-to-Bac system (Life Technologies) was used to produce recombinant bacmid DNA, which was transfected into Sf9 cells using FuGENE HD (Promega) to generate baculovirus. The baculovirus was next used to infect High Five cells (Life Technologies, B85502) at an MOI of 5 to 10. High Five cells were incubated at 28 °C, 110 r.p.m. for 72 h for HA expression. The recombinant HA was purified by Ni-NTA resin, followed by size exclusion chromatography, buffer exchanged into 20 mM Tris, 150 mM NaCl, pH8.0, and concentrated.

<u>Generation of multiplexed coronavirus antigen beads</u>

Streptavidin beads pre-labelled with individual intensities of phycoerythrin (PE)-channel fluorophore (Spherotech, SVFA-2558-6K and SVFB-2558-6K) or fluorescein (FITC)-channel fluorophore (Spherotech, SVFA-2552-6K and SVFA-2552-6K) were incubated with 10 μg/mL each of a subset or all of the following biotinylated antigens: recombinant SARS-CoV-2 spike (Wuhan 1), SARS-CoV-2 RBD, SARS-CoV-2 NTD, SARS-CoV-1 spike and SARS-CoV-1 RBD, MERS-CoV spike, OC43-CoV spike, CCoV-HuPn-2018 spike, pPDCoV-0081-4 spike, HCoV-NL63 spike, HCoV-229E spike, HCoV-HKU1 spike, H1 HA and recombinant CD4 (gifted by Gavin Wright, (*49*)). Antigens acquired in the His-tagged, unbiotinylated form were incubated with 2μg/mL anti-His biotin (Invitrogen, MA1-21315-BTIN) for 20 min at room temperature before being used to label the streptavidin beads. After incubation with the antigens, beads were washed with 0.05% bovine serum albumin (BSA) w/v in phosphate-buffered saline (PBS) and incubated with 10 μg/mL of CD4 to block excess streptavidin sites. The blocked beads were washed twice with 0.05% BSA w/v in PBS and mixed to generate multiplexed configurations as required.

<u>Plasma IgG reactivity to human coronaviruses and donor selection</u>

Multiplexed beads for SARS-CoV-2, SARS-CoV-1, MERS-CoV, HCoV-OC43, HCoV-HKU1, HCoV-229E and HCoV-NL63 spike proteins, as well as CD4 as a negative control, were incubated with donor plasma diluted at 1/50, 1/250 or 1/1250 for 30 min at room temperature, then washed

and stained with 2.5 μg/mL goat anti-human IgG Alexa Fluor 647 (Jackson ImmunoResearch, 109-606-170). Samples were acquired on the iQue Screener Plus (Intellicyt) high-throughput flow cytometer and data were analysed with FlowJo (Version 10.8.1., Ashland, OR). Plasma reactivity was analyzed by calculating area under the curve (AUC) for the IgG binding titration curves and reported after correction using the AUC of the negative control CD4 population. All AUC analyses were performed with GraphPad Prism (Version 9.3.1, San Diego, CA) 19 donors were selected for further analysis if positive for plasma reactivity to the spike proteins of SARS-CoV-2 and at least one other beta-coronavirus.

Memory B cell isolation from PBMCs

Cryopreserved PBMCs were thawed and stained with the following panel: 4',6-diamidino-2-phenylindole (DAPI) (BD564907), CD14-brilliant violet (BV)510 (BioLegend, 301842), CD3-BV510 (BioLegend, 317332), CD56-BV510 (BioLegend, 318340), CD19-Electron Coupled Dye (ECD) (Beckman Coulter, IM2708U), CD21-BV711 (BD, 563163), immunoglobulin A (IgA)-Alexa Fluor 647 (Jackson ImmunoResearch, 109-606-011), IgD-PE-Cy7 (BD, 561314), and immunoglobulin M (IgM)-Peridinin Chlorophyll (PerCP)-Cy5.5 (BD, 561285), CD27-Alexa Fluor 488 (BioLegend, 393204) and CD38-allophycocyanin (APC)-Cy7 (BioLegend, 303534). The cells were sorted using the BD FACSAria IIIu in a Biosafety Level 3 (BSL3) facility and gated on live $CD19^{+}CD14^{-}CD3^{-}CD56^{-}IgM^{-}IgD^{-}IgA^{+}/IgA^{-}$ (memory B cells).

Optofluidic-based isolation of individual cross-reactive antibody secreting B cells

Sorted memory B cells (MBCs) ($CD19^{+}$ IgA^{+}/IgA^{-}) were resuspended with irradiated 3T3-CD40L feeder cells (*50, 51*) in Iscove's Modified Dulbecco's Medium (IMDM) (Gibco, 31980-030) supplemented with 10% heat-inactivated (HI)-fetal bovine serum (FBS) (Gibco, 10438-026), 100 ng/mL IL21 (Gibco, PHC0211), 0.5 μg/mL R848 (Invivogen, tlrl-r848) and 1× Mycozap (Lonza, VZA-2021). The cell suspension was seeded at a density of 50-100 MBCs and 3000 irr-3T3s per well of 384-well plates and co-cultured for 10 days to allow for MBC expansion and activation of Ig secretion. At day 9, culture supernatants were screened for IgA/IgG reactivity against beads coated with 10 μg/mL SARS-CoV-2, SARS-CoV-1, MERS-CoV, OC43-CoV, HKU1-CoV, 229E-CoV and NL63-CoV spike using the iQue screener. On day 10, MBCs from wells of interest were pooled, washed in magnetic-activated cell sorting (MACS) buffer (PBS supplemented with

0.5% w/v BSA and 2mM ethylenediaminetetraacetic acid (EDTA)) and approximately 23,000 cells were loaded onto an OptoSelect 11k chip. Optical Electro-Positioning (OEP) light cages were applied to sort single B cells into nanoliter-volume pens (nanopens) on the chip and antibody secreting cells were screened for cross-reactivity in a two-step assay. First, 7 μm streptavidin beads (Spherotech, SVP-60-5) coated in tandem with 10 μg/mL MERS-CoV spike and 10 μg/mL OC43-CoV spike were re-suspended in a cocktail of 2.5 μg/mL goat anti-human IgG-Alexa Fluor 647 (Jackson ImmunoResearch, 109-606-170) and goat anti-human IgA-cyanine 3 (Cy3) (Jackson ImmunoResearch, 109-166-011), and immobilized in the channels of the OptoSelect 11k chip. Binding of secreted antibody to the beads was detected in the CY5 or TRED channels by capturing images at 6 min intervals over a 30 min time course. In the second step of the assay, the MERS/OC43 beads were replaced with 7 μm streptavidin beads coated with 10 μg/mL SARS-CoV-2 spike, and antibody binding was detected as before. OEP light cages were applied to export individual cross-reactive monoclonal antibody (mAb)-secreting B cells directly into Dynabeads mRNA DIRECT lysis buffer (Life Technologies, 61011) in 96-well plates. Plates were sealed with Microseal foil film (BioRad, MSF1001) and immediately frozen on dry ice before transferring to -80 °C for long-term storage.

mAb sequence analysis and expression

Heavy and light chain sequences were amplified from single B cell lysates using RT-PCR (*20, 43, 52*) and resolved by Sanger Sequencing (Eurofins and Genewiz). Analyses of the heavy chain variable (VH) and (lambda/kappa variable) Vλ/Vκ genes, complementarity-determining region 3 (CDR3) sequences, and percentage of somatic mutations were carried out using Geneious Prime (Version 2021.0.3, https://www.geneious.com) and the International Immunogenetics Information System database (IMGT, http://www.imgt.org/) (*53*). Matched pairs of antibody VH and Vλ/Vκ sequences were commercially cloned into plasmids containing an IgG1 or relevant light chain backbone and expressed as recombinant antibody (Genscript). For germline reversion, the V and J sequences of the antibody heavy and light chains were reverted to those of the closest alleles based on the IMGT database. mAbs were also expressed in-house by transient transfection of Expi293 cells (Gibco, A14527) using the ExpiFectamine 293 Transfection Kit (Gibco, A14524) according to the manufacturer's instructions. Recombinant IgG mAbs were purified using HiTrap Protein A columns (Cytiva/GE Healthcare Life Sciences, 17040303).

Human leukocyte antigen (HLA) typing for donor identification

Several mAbs were isolated from screens that involved pooling of B cells from two different individuals. To identify the source donor of these mAbs, a commercially available ScisGo®-HLA-v6 kit (Scisco Genetics Inc., Seattle WA) employing an amplicon-based sequencing by synthesis approach was used to determine HLA types of amplified complementary DNA (cDNA) from single cell isolates. The approach uses a two-stage amplicon-based PCR for locus amplification and sample barcoding. Although designed for amplification from genomic DNA, a subset of the kit amplicons was functional in amplifying product from cDNA. Briefly, samples were sequentially applied to stage 1 (S1) and stage 2 (S2) PCR amplification according to the manufacturer supplied protocol. After amplification, the reactions were combined, purified, and applied to a MiSeq using Illumina Version 2 chemistry with 500-cycle, paired-end sequencing (Illumina, San Diego, CA). Data assembly and analysis were performed using Sciscloud® (Scisco Genetics Inc., Seattle WA) computational tools adapted specifically to assemble HLA genomic sequences derivative from the ScisGo®-HLA-v6 kit. Amplified portions of the HLA class I and class II genes were compared with prior typing data allowing the unambiguous identification of corresponding samples. Access to all software for data transfer and analysis was included as a component of the kit and made available through a web browser.

Recombinant mAb binding to coronavirus antigens

Recombinant mAbs were diluted 4-fold in 0.05% BSA w/v in PBS to generate a 47.7 ng/mL – 200 µg/mL dilution series. Multiplexed antigen-labelled beads were incubated with mAb titrations for 30 min at room temperature, then washed and stained with 2.5 µg/mL goat anti-human IgG Alexa Fluor 647 (Jackson ImmunoResearch, 109-606-170). Samples were acquired on the iQue Screener Plus and data were analysed with FlowJo. Data points from the titration curves were interconnected without logistic regression and AUC analyses were performed with GraphPad Prism and reported after correction using the AUC of the negative control CD4 population.

Phylogenetic tree generation

Full-length amino acid sequences of SARS-CoV-2 (accession #NC_045512.2), SARS-CoV (accession # AY278741.1), MERS-CoV (accession # NC_019843), HCoV-NL63 (accession

#NC_005831.2), HCoV-229E (accession #NC_002645.1), CCoV HuPn-2018 (accession #MW591993.2) and PDCov-0081-4 (accession #MW685622) were aligned using the L-INS-i method of MAFFT version 7 (*54*). A Neighbor-Joining tree based on the sequence alignments was constructed in MEGA11 with 500 bootstrap resamplings. The phylogenetic tree was visualized in the Interactive Tree of Life (iTOL) online server (*55*).

Sequence conservation

Full-length amino acid sequences of spike from SARS-CoV-2 Wuhan-Hu-1 (Genbank accession # YP_009724390), SARS-CoV-2 B.1.1.7 (Genbank accession # QWE88920), SARS-CoV-2 B.1.351 (accession # Genbank QRN78347), SARS-CoV-2 P.1 (Genbank accession # QVE55289), SARS-CoV-2 B.1.617.2 (Genbank accession # QWK65230), SARS-CoV-2 BA.1 (Genbank accession # UFO69279), SARS-CoV-2 BA.2 (Genbank accession # UJE45220), SARS-CoV-2 BA.2.12.1 (Genbank accession # UMZ92892), SARS-CoV-2 BA.4 (Genbank accession # UPP14409), Avian Infectious Bronchitis Virus (Uniprot accession # F4MIW6), BatCoV-HKU3 (Uniprot accession # Q3LZX1), BatCoV-HKU4 (Uniprot accession # A3EX94), BatCoV-HKU9 (Genbank accession # ABN10911), BatCoV-RaTG13 (Genbank accession # QHR63300), BatCoV-WIV1 (Genbank accession # AGZ48831), WhaleCoV-SW1 (Uniprot accession # B2BW33), BuCoV-HKU11 (Genbank accession # ACJ12044), CCoV-HuPn-2018 (Genbank accession # QVL91811), Civet-SARS-CoV-007/2004 (Genbank accession # AAU04646), HCoV-229E (Genbank accession # NP_073551), HCoV-HKU1 N5 (Uniprot accession # Q0ZME7), HCoV-NL63 (accession # Genbank YP_003767), HCoV-OC43 (Uniprot accession # P36334), MERS-EMC/2012 (Genbank accession #YP_009047204) , MuCoV-HKU13-3514 (Uniprot accession # B6VDY7), Pangolin-CoV-GX/P2V (Genbank accession # QIQ54048), PDCoV/Haiti/Human/0081-4/2014 (Genbank accession # QWE80492), PDCoV/Haiti/Human/0329-4/2015 (Genbank accession # QWE80508), SARS-CoV-Urbani (Genbank accession # AAP13441.1), Mouse Hepatitis Virus (Uniprot accession # P11224), ThCoV-HKU12 (Uniprot accession # B6VDX8), TCoV (Uniprot accession # B3FHU5), WiCoV-HKU20 (Uniprot accession # H9BR25) were aligned using MAFFT v7 server using a BLOSUM62 scoring matrix and L-INS-i algorithm. The sequence alignment was used to generate a sequence logo plot using the Weblogo 3.0 server (*56*)and to color conserved amino acid residues on a pre-fusion stabilized spike protein (PDB 6VSB) using Chimera X.

SARS-CoV-2 spike and S2 subunit epitope binning by surface plasmon resonance (SPR)

Epitope binning experiments were performed on the Carterra LSA with cross-reactive mAbs coupled to an HC30M chip (Carterra). The chip was conditioned by successive injections of 50 mM NaOH, 500 mM NaCl and 10 mM glycine pH 2, then primed with 2-(N-morpholino)ethanesulfonic acid (MES) supplemented with 0.05% Tween. To prepare the mAb array, the chip was activated with a 1:1 mixture of 400 mM 1-Ethyl-3-(3-dimethylaminopropyl)carbodiimide (EDC) and 100 mM N-hydroxysuccinimide (NHS) (ThermoFisher Scientific) followed by direct coupling of 0.1 mg./mL, 1.0 µg/mL and 10 µg/mL of the mAbs in pH 4.5 acetate buffer onto discrete spots on the chip. Excess binding sites on the chip were blocked with 1M ethanolamine, pH 8.5. For pre-mixed binning experiments with trimeric SARS-CoV-2 spike, 20 nM SARS-CoV-2 spike was pre-mixed in a 1:1 volume ratio with 2 µM of each sandwiching antibody and the mAb-spike complexes were then injected onto the array. For classical binning experiments with SARS-CoV-2 S2 domain, 200 nM of monomeric SARS-CoV-2 S2 domain was directly injected onto the chip followed by 10 µg/mL of each sandwiching mAb. The chip was regenerated with 10 mM glycine pH 2.0 after each sandwiching antibody injection. Binning data were analyzed using the Epitope Software (Carterra).

SARS-CoV-2 S2 binding kinetics with Fab fragments of cross-reactive antibodies

Fab fragments were generated using the Pierce Fab Preparation kit (ThermoFisher Scientific, 44985) according to the manufacturer's instructions. Briefly, 250 mg to 500 mg of mAb diluted in PBS were buffer exchanged into digestion buffer (20 mM cysteine-HCl) using a 7K molecular weight cutoff (MWCO) Zeba desalting column. Immobilized papain was transferred to a spin-column and equilibrated with 0.5 mL digestion buffer. mAbs were cleaved by papain for 3 hours at 37C in an end-over-end mixer. Digested Fabs were purified using the Protein G HP SpinTrap column (Cytiva, 28903134) and desalted to remove excess cysteine present following the digestion. Following chip activation, Fabs were coupled to an HC-30M chip (Carterra) at a concentration of 1.67 µg/mL. Serial dilutions of SARS-CoV-2 Wuhan Hu-1 S2P or S2 were injected onto the chip and kinetics was measured using a 10 min association and 20 min dissociation followed by regeneration with 10 mM glycine pH 2.0. Data were analyzed using the

Kinetics Software (Carterra), including bulk shift adjustments to account for changes in buffer refractive index.

SARS-CoV-2 S2 peptide mapping

Peptides spanning the entire SARS-CoV-2 S2 domain (Ser686- Lys1211, Accession #YP_009724390.1) were commercially synthesized (JPT Peptide Technologies). Each peptide was 15 amino acids in length, overlapped its flanking peptides by 12 residues and carried an N-terminal biotin tag followed by a Ttds (Trioxatridecan-succinamic acid) linker. A further 8 irrelevant oligomers representing random H1 hemagglutinin peptides were included in the panel as negative controls. The lyophilized biotinylated peptides were reconstituted to 1 mg/mL in dimethyl sulfoxide (DMSO), then further diluted in HEPES-buffered saline-Tween-EDTA (HBSTE) supplemented with 0.05% BSA and directly coupled to the streptavidin surface of a SAD200M chip (Carterra) at 0.1 µg/mL and 10 µg/mL. Broadly reactive mAbs were successively injected onto the peptide array at 10 µg/mL and binding data were acquired over a 5 min association phase and 1 min dissociation phase using the Carterra LSA. 3 successive injections of 10 mM glycine pH 2.0 were used to regenerate antibody binding sites on the arrayed peptides after each antibody injection. Data were analyzed using the Epitope Software (Carterra).

Imaging-based fusion inhibition assay

HeLa cell lines (Expasy CVCL_0030) stably expressing either CoV spike proteins or their cognate receptor were generated as previously reported (*15*) and maintained in Dulbecco's Modified Eagle Medium (DMEM) + 10% FBS + 1% Pen/Strep + 1% Glutamax. To generate spike expressing cells, HeLa cells were transduced with lentivirus encoding both the nuclear localization signal (NLS)-red fluorescent protein (RFP) and a relevant CoV spike protein, stained with corresponding mAbs and sorted to collect the $RFP^{high}/Spike^{high}$ population. To generate green fluorescent protein (GFP)-tagged receptor cell lines, HeLa-angiotensin-converting enzyme 2 (ACE2) cells were transduced with lentivirus encoding GFP and sorted to collect the $GFP^{high}/ACE2^{high}$ population. For fusion inhibition assays, 5000 $RFP^{+}/Spike^{+}$ HeLa cells were seeded per well in 96-well plates one day before experiment and cultured overnight. mAbs were added to the wells at a final concentration of 200 µg/mL and cultures were further incubated at 37 ^{o}C for 1h. 8,000 $GFP^{+}/ACE2^{+}$ HeLa cells were then added to each well and the co-cultures were maintained

overnight to allow for syncytia development. After visual confirmation of syncytia under the microscope, the culture medium was replaced with 4% paraformaldehyde (PFA) and cells were fixed for 15 min and washed twice with PBS. Fixed cultures were counter-stained with 1 μg/mL Hoechst for 10 min and washed twice with PBS. Images were acquired in A488, A568 and DAPI channels using a BZ-X fluorescence microscope (KEYENCE) and processed using Fiji ImageJ (*57*).

Colorimetric fusion inhibition assay

HEKBlue cells (Invivogen, hkb-hace2tpsa) stably transfected to express human angiotensin-I-converting enzyme-2 (hACE2), human transmembrane protease serine 2 (TMPRSS2), and an NF-kB inducible secreted embryonic alkaline phosphatase (SEAP) reporter were used as “acceptor cells”. While 293-hMyD88 cells (Invivogen, 293-hmyd) stably transfected to express hMyD88 were used as "donor cells”. Both cell lines were purchased commercially and cultured per the manufacturer’s instructions. All incubations were performed at 5% CO_2 and 37°C. To measure inhibition, 9 x 10^5 donor cells were prepared in growth media - DMEM with 4.5 g/L glucose, 4 mM L-glutamine (Gibco, 10566016) supplemented with heat-inactivated 10% fetal bovine serum, 100 U/mL Penicillin and Streptomycin (Gibco 15140122), 100 μg/mL Normocin (Invivogen, ant-nr) and seeded in 6-well tissue culture treated plates (Corning) overnight. 18.75 μg/mL of spike plasmids or vector controls were complexed at a 1:3 ratio with PEI Max for 20 minutes, added dropwise to donor cells, and incubated overnight. Donor cells were harvested using 1x PBS (Lonza) and 5 x 10^4 cells were mixed 1:1 with 3-fold serial dilution of each antibody. After 1 hour, 5 x 10^4 acceptor cells were added to each well and incubated overnight. To measure SEAP expression, Quantiblue substrate (Invivogen, rep-qbs) was prepared as described by the manufacturer. 100 μL of substrate was mixed with 100 μL of supernatant and incubated for 3 hours. Absorbance was measured using an EnSpire Multimode (Perkin Elmer) plate reader at 635 nm. Percent inhibition was calculated as described in (*58*), briefly (1-(E-N)/(P-N)) x 100; where “E” is absorbance of the antibody treatment group, “N” is the absorbance of vector control, and “P” is the absorbance of the no antibody treatment group.

Shotgun mutagenesis epitope mapping of antibodies by alanine scanning

Epitope mapping was performed essentially as previously described (*59*), using a SARS-CoV-2 (Wuhan Hu-1 strain) S2 subunit shotgun mutagenesis mutation library, made using a full-length expression construct for the SARS-CoV-2 spike glycoprotein. 513 S2 residues (between residues 689 -1247) were mutated individually to alanine, and alanine residues to serine. Mutations were confirmed by DNA sequencing, and clones arrayed in a 384-well plate, one mutant per well. Binding of mAbs to each mutant clone in the alanine scanning library was determined, in duplicate, by high-throughput flow cytometry. A plasmid encoding cDNA for each spike protein mutant was transfected into HEK-293T cells and allowed to express for 22 h. Cells were fixed in 4% (v/v) PFA (Electron Microscopy Sciences), and permeabilized with 0.1% (w/v) saponin (Sigma-Aldrich) in PBS before incubation with mAbs diluted in PBS, 10% normal goat serum (Sigma), and 0.1% saponin. mAb screening concentrations were determined using an independent immunofluorescence titration curve against cells expressing wild-type spike protein to ensure that signals were within the linear range of detection. Antibodies were detected using 3.75 μg/mL of Alexa-Fluor-488-conjugated secondary antibodies (Jackson ImmunoResearch) in 10% normal goat serum with 0.1% saponin. Cells were washed three times with PBS/0.1% saponin followed by two washes in PBS, and mean cellular fluorescence was detected using a high-throughput Intellicyt iQue flow cytometer (Sartorius). Antibody reactivity against each mutant spike protein clone was calculated relative to wild-type spike protein reactivity by subtracting the signal from mock-transfected controls and normalizing to the signal from wild-type spike-transfected controls. Mutations within clones were identified as critical to the mAb epitope if they did not support reactivity of the test mAb but supported reactivity of other SARS-CoV-2 antibodies. This counter-screen strategy facilitates the exclusion of spike protein mutants that are locally misfolded or have an expression defect.

Expression and purification of Fabs for structural studies

Sequences of the variable domains of the heavy chain and light chain of COV44-62, COV44-79, and COV91-27 were codon optimized (Genscript) and fused with an N-terminal secreting signal peptide, and a human Fab expressing vector. The three Fabs were expressed by co-transfection of plasmids of heavy and light chain at the ratio of 2:1 in ExpiCHO expression system (Life Technologies) for two weeks according to the manufacturer's manual. Supernatants were

harvested, purified with CaptureSelect CH1-XL resin (Life Technologies), and followed by size exclusion chromatography (SEC) in 20 mM Tris buffer with 150 mM NaCl at pH 7.4 (TBS). Fabs were concentrated to at least 10 mg/ml before crystallization trials.

Crystallization and structural determination

Fusion peptides were synthesized by GenScript. The complex of each Fab with peptide was formed by mixing each Fab with a 10-fold molar ratio of peptide and incubating overnight at 4°C without further purification. Each complex was adjusted to ~10 mg/ml in TBS buffer, pH 7.4. The complexes were screened for crystallization on the robotic high-throughput CrystalMation system (Rigaku) at The Scripps Research Institute using the JCSG Core Suite (QIAGEN) as precipitant. Crystallization trials were setup by the vapor diffusion method in sitting drops containing 0.1 µl of protein and 0.1 µl of reservoir solution. The optimized crystallization condition for COV44-62 with fusion peptide was 0.1 M sodium citrate, pH 4, 1 M lithium chloride, and 10% polyethylene glycol (PEG)6000. The optimized condition for COV44-79 with fusion peptide was 0.1 M Tris, pH 8.5, 0.01 M nickel (II) chloride, and 20% PEG monomethyl ether 2000 and, for the COV91-27-peptide complex, was 70% 2-methyl-2,4-pentanediol and 0.1M HEPES, pH 7.5. Crystals were harvested on or before day 14 and then soaked in reservoir solution containing 20% (v/v) ethylene glycol as cryoprotectant for COV44-62 and COV44-79 complexes, and 15% (v/v) ethylene glycol for the COV91-27-peptide complex. The harvested crystals were then flash-cooled and stored in liquid nitrogen until data collection. Diffraction data were collected at cryogenic temperature (100 K) at the Stanford Synchrotron Radiation Lightsource on Scripps/Stanford beamline 12-1 with a beam wavelength of 0.97946 Å for the COV44-62 and COV91-27 complexes, and at beamline 23-ID-B of the Argonne Photon Source (APS) with a beam wavelength of 1.033167 Å for the COV44-79 complex. The diffraction data were processed with HKL2000 (*60*). Structures were solved by molecular replacement using Phaser (*61*) with the models generated by Repertoire Builder (https://sysimm.org/rep_builder/) for COV44-62, COV44-79, and COV91-27. Iterative model building and refinement were carried out in Coot (*62*) and PHENIX (*63*), respectively. Buried and accessible surface areas were calculated with PISA (*44*).

Authentic OC43-CoV-GFP virus propagation and neutralization assay

Rhabdomyosarcoma cells (RD, ATCC CCL-136) were maintained at 37°C and 5% CO_2 in no-glucose DMEM (Gibco, 11966-025), supplemented with 10% HI-FBS, 4500 mg/mL glucose, 1 mM sodium pyruvate (Gibco, 11360-070), 1 mM HEPES (Gibco, 15630-080) and 50 µg/mL gentamycin (Quality Biological, 120-098-661). RD cells were seeded into a T225cm^2 flask and cultured to achieve 90% confluency. Cell cultures were inoculated with infectious GFP-tagged HCoV-OC43 at 0.01 MOI in FBS-free, high-glucose DMEM supplemented with 1X GlutaMax (Gibco, 11965-092) and sodium pyruvate. Cultures were maintained at 35°C for 1 h with gentle rocking every 10 min. The inoculum was replaced with prewarmed high glucose DMEM supplemented with 1× Glutamax, 1× non-essential amino acids (Gibco, 12491-015), 2% HI-FBS, 15 mM HEPES and 50 µg/mL gentamicin, and the culture was further maintained for 3-4 days at 35˚C and 5-9% CO_2. To harvest progeny virions, the virus-containing culture media was cleared at 234 × g for 30 min at 4˚C, and the cleared supernatant was aliquoted and stored at -80˚C. The volume of OC43-GFP virus needed to achieve 75% infection ($TCID_{75}$) of RD cell cultures was determined by endpoint dilution. For neutralization assays, 5 × 10^4 RD cells were inoculated at $TCID_{75\%}$ OC43-GFP virus and incubated for 1h at 35°C. 4-fold serial dilutions (73 ng/mL - 300 µg/mL) of each mAb were incubated with $TCID_{75}$ OC43-GFP virus for 1h at 35°C. 60 µL of mAb-virus mixture was used to inoculate each well containing 5 × 10^4 RD cells and cultures were incubated for 24 h at 35°C. GFP expression was measured on the iQue Screener Plus and analysed using FlowJo. %Neutralization was determined by (100 × (1-(GFP_x Min_{GFP}) / ($Max_{GFP}Min_{GFP}$)), where uninfected, untreated cells = Min_{GFP} and untreated, infected cells = Max_{GFP}.

Pseudovirus production and neutralization assays ($Assay_{NIH}$)

Codon-optimized cDNA encoding full-length spike from SARS-CoV-2 (GenBank ID: QHD43416.1), SARS-CoV (Urbani_ GenBank: AAP13441.1), MERS-CoV EMC_ GenBank: AFS88936), HCoV-NL63 (GenBank: Q6Q1S2.1) and HCoV-229E (GenBank: AOG74783.1) were synthesized (Genscript), cloned into the mammalian expression vector VRC8400 (*64*) and confirmed by sequencing. These full-length spike plasmids were used for pseudovirus production. Spike-containing lentiviral pseudovirions were produced by co-transfection of packaging plasmid pCMVdR8.2, transducing plasmid pHR' CMV-Luc, a TMPRSS2 plasmid and full-length spike plasmids from SARS-CoV-2, SARS-CoV, MERS-CoV, HCoV-NL63 and HCoV-229E into 293T

cells (ATCC CRL-11268) using Lipofectamine 3000 transfection reagent (ThermoFisher Scientific, Asheville, NC, L3000-001) (*65*). 293 flpin-TMPRSS2-ACE2 cells (provided by Dr. Adrian Creanga, VRC/NIH) were used for SARS-CoV-2, SARS-CoV and hCoV-NL63 while HuH7.5 cells (provided by Dr. Deborah R. Taylor) (*66*) were used for MERS-CoV and hCoV-229E neutralization assay. Cells were plated into 96-well white/black Isoplates (PerkinElmer, Waltham, MA) at 10,000 cells per well the day before infection of pseudovirus. Serial dilutions of mAbs were mixed with titrated pseudovirus, incubated for 45 min at 37°C and added to cells in triplicate. Following 2 h of incubation, wells were replenished with 150 ml of fresh media. Cells were lysed 72 h later, and luciferase activity was measured with Microbeta (Perkin Elmer). 50% neutralization titers (NT_{50}) were calculated using the dose-response-inhibition model with 5-parameter Hill slope equation in GraphPad Prism 9.

Pseudovirus production and neutralization assays ($Assay_{Scripps}$)

Lentiviral based pseudo-viruses were produced similarly to a previous report (*42*). HEK293T cells (Expasy CVCL_0063) were seeded in 6 well plates and grown to ~80% confluency in DMEM (Lonza, 12-614F) with P/S, glutamine and 10% heat-inactivated FBS. 2.5μg 2nd generation lentivirus backbone plasmid pCMV-dR8.2 dvpr (Addgene #8455), 2μg pBOBI-FLuc (Addgene #170674) and 1μg truncated coronavirus spike expressing plasmids (SARS: Addgene #170447; SARS2 #170442; MERS #170448; NL63 #172666; alpha strain #170451; beta #170449; gamma #170450; delta #172320; BA.1 180375; BA.2 #183700; BA.4/5 #186810) were co-transfected in HEK293T with Lipofectamine 2000 (ThermoFisher Scientific, 11668019) to produce single-round infection-competent pseudoviruses. The medium was changed 12-16 hours post transfection. Pseudovirus-containing supernatants were collected 48 and 72 hours post transfection, centrifuged at 1,500 × g for 10 min and the viral titers were measured by luciferase activity in relative light units (RLU) (Bright-Glo Luciferase Assay System, Promega, E2620). The supernatants are aliquoted and stored at -80˚C until further use. Pseudotyped viral neutralization assays were performed similar to a previous report (*42*). 20μL pseudovirus supernatant were added into 20 μL serial dilutions of purified antibodies (starting from 100 μg/mL and dilute by 3-fold) in 384-well plates (Corning 3570). The mixture was incubated for one hour at 37°C, after which 5,000 HeLa-hACE2 cells/ well (in 20 μL medium containing 30 μg/ml Dextran) were directly added to the mixture. After incubation at 37°C for 42-48 h, the medium was aspirated and luciferase activity

was measured by adding 25 μL 1× luciferase substrate. Neutralizing activity was calculated by reduction in luciferase activity compared to the virus controls. 50% neutralization titers (NT_{50}) were calculated using the dose-response-inhibition model with 5-parameter Hill slope equation in GraphPad Prism 9.

Authentic SARS-CoV-2 neutralization assay

One day prior to infection, Vero E6 cells (Expasy CVCL_XD71) were seeded in 96-well half-well plates at 10,000 cells/well in a volume of 50 μL complete DMEM medium (supplemented with 10% heat-inactivated serum, 1% GlutaMAX, 1% P/S). Serially diluted antibodies and SARS-CoV-2 (diluted to a final concentration of 1,000 plaque forming units/well) and were mixed 1:1 in a total volume of 50 μL, incubated for 30 minutes, and added to the cells (performed in duplicate). The cells were incubated at 37°C for 24 hours. The medium was removed and disposed of appropriately. The cells were fixed by immersing the plate into 4% paraformaldehyde for 1 hour and subsequently washing 3 times with PBS. The plate was stored at 4°C or gently shaken for 30 minutes with 100 μL/well of permeabilization buffer (PBS with 1% Triton-X). All solutions were removed, 100 μL of 3% BSA was added and the plate was incubated for 2 hours at RT. A mixture of primary antibodies including equal amounts of CC6.29, CC6.33, L25-dP06E11, CC12.23, CC12.25 were diluted in PBS with 1% BSA to a final concentration of 2 μg/mL and 50 μL of this cocktail was added to each well. After 1h incubation at RT, the solutions were discarded, and the plates were washed 3 times with PBST (PBS + 0.05% Tween-20). 50 μL of 1 ug/mL HRP-conjugated goat anti-human IgG (Invitrogen, A18817) diluted in PBS w/ 1% BSA was added to each well and the plate was incubated at RT for 1h. The plate was washed with PBST for at least 5 times and pressed on dry tissue paper before adding 50 μL of POD substrate to each well (Roche, 11582950001) according to the manufacturer's instructions. Chemiluminescence intensity was read in a plate reader. The neutralization percentage was calculated using the following equation:

$$\%Neut = \frac{Read - NC}{PC - NC} \times 100\%$$

Where NC is the average readout of negative controls (wells without live virus), and PC is the average of positive controls (wells with no antibody).

Authentic MERS-CoV neutralization assay

On the day before infection, Vero E6 cells (BEI NR-596) were plated into 384-well tissue-culture treated plates at a density of 6,000 cells per well in 30 µL DMEM (Gibco) with 10% FBS (Sigma). On the day of infection, a serial dilution of antibodies was performed in 384-well deep well plates to generate a twelve-point dose curve (in quadruplicate), with the highest concentration at 200 µg/mL. Under biocontainment conditions, MERS-CoV was diluted in the same medium to a concentration of 18,000 plaque forming units (PFU) per 30 µL (6×10^5 PFU/mL). Next, 30 µL of this virus inoculum was added to the antibody dilution series, resulting in a starting antibody concentration of 100 µg/mL after addition of virus. The virus/antibody mixtures were incubated at 37 C for one hour, following which 30 µL of the mixtures were transferred into the plate with Vero E6 cells for a final assay volume of 60 µL. After 24 hours, 10% neutral buffered formalin was added to the plates for fixation of the samples. The plates were removed from biocontainment and the cells were stained with an anti-MERS-CoV spike antibody (Sino Biological, 40069-R723), followed by a goat anti-rabbit secondary antibody conjugated to Alexa 647 (Life Technologies, A21245). Hoechst stain to the cells was added to allow detection of cell nuclei. Fluorescence emission was analyzed using the Operetta high-content imaging system (PerkinElmer). Half-maximal inhibitory concentration (IC_{50}) was calculated as previously described (*67*) using Prism (GraphPad). Z' factor scores were examined for quality-control of each assay plate.

Hamsterization of human monoclonal antibodies

Genomes corresponding to the mouse IgG2a heavy and light chains were aligned to the genome assembly MesAur1.0 (GCA_000349665.1) for a female Syrian golden hamster downloaded from Genbank. Hamster genes with the highest homology to the mouse IgG2a heavy chain, lambda and kappa light chains genes were cloned into a pCDNA3.4 vector (Genscript) and expressed in Expi293 cells as described above.

Syrian hamster efficacy studies

Approximately 5-6 weeks old Golden Syrian hamsters with equal number of males and females were acquired from Envigo (Indianapolis, IN USA). Hamsters were delivered to the Integrated Research Facility (IRF) ten days prior to study onset for acclimation. Individually housed hamsters were assigned to eight groups (n = 12 each) by a statistician according to weight and gender. The study remained blinded to all other individuals (except the Study Director team that prepared the

antibodies and virus for challenge) until termination of the in-life phase of the study. Comparative Medicine Team remained blinded throughout the course of the in-life portion of the study to prevent bias in clinical scoring. Animal ID's and respective treatments were unblinded once all Core and Pathology data packages were received. Animals were randomly assigned to groups to balance as closely as possible between ages (same in this experiment), weight ranges, and sex distribution prior to challenge in consultation with a NIH statistician.
Antibody products were evaluated in this study with single treatment at a dose of 16 mg/kg (16 mg/kg each of cocktail). PBS-treated (mock-treated) and SARS-CoV-2 naïve (mock-exposed) hamsters were included as controls. Animals were treated with antibody or PBS by intraperitoneal (IP) inoculation 24 hours prior to exposure to 5 log_{10} pfu SARS-CoV-2 (WA01) via intranasal (IN) installation in the prophylaxis study. Animals were weighed prior to study initiation (Day -2) to determine the average weight of each group for calculation of the treatment dose. Following virus inoculation, animals were weighed and observed daily to monitor the clinical signs of disease. Half of the animals in each group were euthanized on day 3 post-infection and the other half of each group was euthanized on day 7. At euthanasia, blood, nasal turbinate and lung tissues were collected for further analysis and necropsies were performed. Lung weight was measured, and the gross pathology scores were assigned by pathologist at the necropsy. None of the animals used in the study reached endpoint criteria that would have required an unscheduled euthanasia.

Animal research was conducted under an IACUC approved protocols (protocol numbers: SARS-CoV-2-HAM-65E-4G Amendment #2, Amendment #3,4) at the Integrated Research Facility, Frederick, Maryland, in compliance with the Animal Welfare Act and other federal statutes and regulations relating to animals and experiments involving animals. The facilities at the IRF where this research was conducted are fully accredited by the Association for Assessment and Accreditation of Laboratory Animal Care, International and adheres to principles stated in the Guide for the Care and Use of Laboratory Animals, National Research Council, 2011.

Plaque assay

Lung samples from hamsters were obtained on day 3 post-infection with SARS-CoV-2. Vero E6 cells were seeded onto 6-well plates at 1×10^6 cells/well to reach at least 90% confluence on the next day. Ten-fold serial dilutions of the samples were added to the wells (in triplicate of 300

µL/well). The samples were incubated for 1 h at 37°C, 5% CO2 with rocking every 15 min. Next, 2 mL of Avicel overlay diluted 1:1 (final concentration 2.5%) in 2× EMEM (Quality Biological) containing 4% FBS was added to the cells (*68*). The cells were incubated at 37°C, 5% CO2 for 48 h. The Avicel overlay was removed and the cells were fixed with 0.2% crystal violet in 900 mL of 10% NBF and 100 mL of 2% Gentian Violet for 30 min at room temperature. The plates were washed with water and the plaques were counted. Viral load data were reported as plaque forming units (PFU)/g of homogenized tissue.

RT-qPCR

Lung samples from hamsters for RNA extraction and RT-qPCR analysis of viral subgenomic RNA were obtained on day 3 post-infection with SARS-CoV-2 and treated with TRIzol LS (Thermo Fisher Scientific) according to the manufacturer's instructions. Briefly, 70 µL of TRIzol LS-inactivated sample was mixed with 280 µL of Buffer AVL (QIAGEN) containing 3 µg of carrier RNA (polyA, supplied with QIAamp Viral RNA Mini kit, QIAGEN). RNA was extracted using the QIAamp Viral RNA Mini Kit (QIAGEN), following the manufacturer's protocol. The samples were eluted in 70 µL of Buffer AVE, divided into two portions of equal volume and kept at -80°C until needed for the RT-qPCR assay. They were quantified using the 2019-Novel Coronavirus (2019-nCoV) Real-time RT-PCR protocol established by the CDC was used to quantify SARS-CoV-2 viral RNA targets in the orf1a gene. An ABI 7500 FastDx (Applied Biosystems) was used for the assay, following manufacturer guidelines. PCR cycle conditions were as follows: 50°C for 5 min, 95°C for 20 s, 95°C for 3 s, 60°C for 30 s, with the final 2 steps repeated for 45 cycles. Serially 10-fold diluted copies of DNA (from 9 log10 to 0 log10 vRNA) made of the SARS-CoV-2 RT-qPCR assay target were constructed as gBlocks (Integrated DNA Technologies, Coralville, IA, USA) and used to generate a standard curve to determine quantifiable Ct values. The ABI 7500 software version 1.4.1 (Applied Biosystems) was used to determine the viral RNA copy numbers in each sample, with data reported as viral RNA copies per mg of lung tissue.

Vaccinee and convalescent plasma binding to peptides

Polyclonal IgG antibodies from plasma or sera of vaccinated, convalescent, or naïve donors were purified using the Pierce Protein G Spin Plate (ThermoFisher Scientific, 45204). Briefly, plasma or sera were diluted 1:4 in PBS and incubated with Protein G for 30 min, 600 rpm at room

temperature. The flowthrough was collected and incubated with the Protein G resin for 15 min to ensure maximal binding. The Protein G resin was washed four times with PBS and the IgG was eluted with Protein G Elution Buffer pH 2.0 (ThermoFisher Scientific, 21028) and neutralized with 1 M Tris pH 8.0 (Corning 46-031-CM). Purified IgG was desalted using a 40 kDa MWCO Zeba Plate (ThermoFisher Scientific 87774) and diluted to 100 µg/mL to assess epitope reactivity. The polyclonal IgG was added to a SAD200M chip (Carterra) that was previously coated with biotinylated peptide 43 as described above. IgG binding was analyzed using the Epitope Software (Carterra).

Statistical analyses

Neutralization and fusion inhibition curves were fitted using the dose-response-inhibition model of non-linear regression analysis with 5-parameter Hill slope equation. For neutralization assays, 50% antibody neutralization titers (NT50) values were interpolated from the resulting curves. Statistical significance for average body weight was analyzed across the 7-day time-course using a mixed-effects repeated measures model with Dunnett's post-test multiple comparison. Statistical analyses for hamster pathology scores and viral loads were analyzed by Kruskal-Wallis tests with Dunn's post-test multiple comparison for multiple groups or Mann-Whitney U-test for comparisons of two groups. For all analyses $*P < 0.05$, $**P < 0.01$, $***P < 0.001$, $****P < 0.0001$ and ns, not significant. Binding of polyclonal IgG from vaccinated and convalescent donors to the fusion peptide was analyzed using a nested, mixed-model ANOVA with Bonferroni-adjusted post hoc comparisons. Descriptive statistics and statistical analyses were performed using Prism version 9.3.1 (GraphPad). Phylogenetic bootstrap resampling was run with 500 iterations in MEGA11. Data for authentic MERS-CoV and SARS-CoV-2 neutralization, $Assay_{NIH}$ pseudovirus neutralization, SPR-based kinetic studies and shotgun alanine mutagenesis were obtained from 1 experiment. Values for $Assay_{Scripps}$ neutralization are reported as the average from two experiments. All other data are representative of at least two experiments.

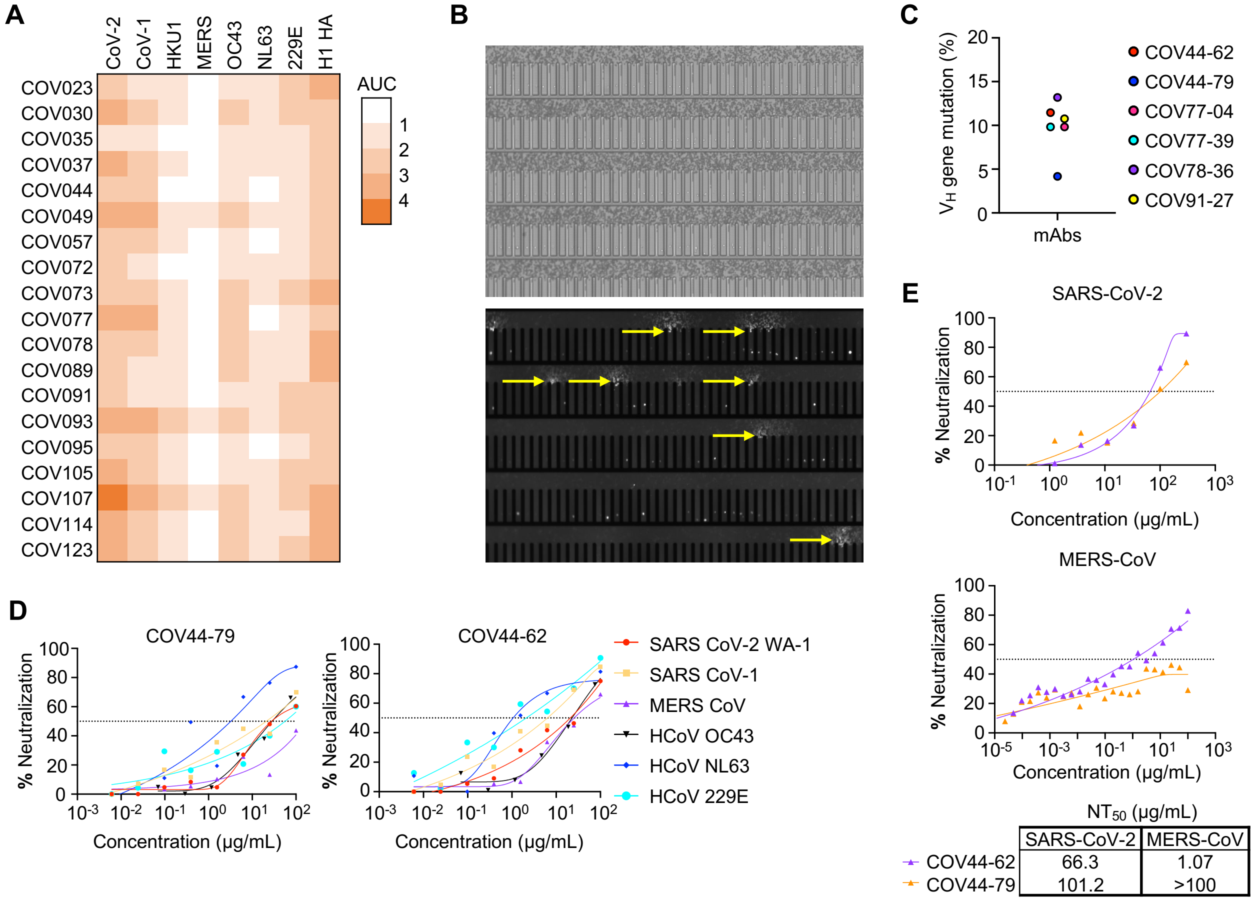

Fig. S1. COVID-19 convalescent donor screening and isolation of broadly neutralizing mAbs. (**A**) COVID-19 convalescent plasma reactivity to the seven human coronaviruses. The heat map represents area under the curve (AUC) values for each antigen after subtraction with values for the negative control antigen CD4. H1 haemagglutinin was included as a control. (**B**) Representative images show optofluidic screening of MBC secreted antibodies for cross-reactivity. The top panel shows individual MBCs sorted into nanopens and the bottom panel shows the fluorescent signal of antigen-specific antibodies binding to coronavirus spike-coated beads (yellow arrows). (**C**) V_H gene mutation levels of fusion peptide-targeting mAbs. (**D**) Neutralization curves of COV44-62 and COV44-79 against authentic HCoV-OC43, as well as SARS-CoV-2 Wuhan Hu-1, SARS-CoV-1, MERS-CoV, HCoV-NL63 and HCoV-229E envelope-pseudotyped virus. **(E)** Neutralization curves of COV44-62 and COV44-79 against authentic SARS-CoV-2 Wuhan Hu-1 and MERS-CoV.

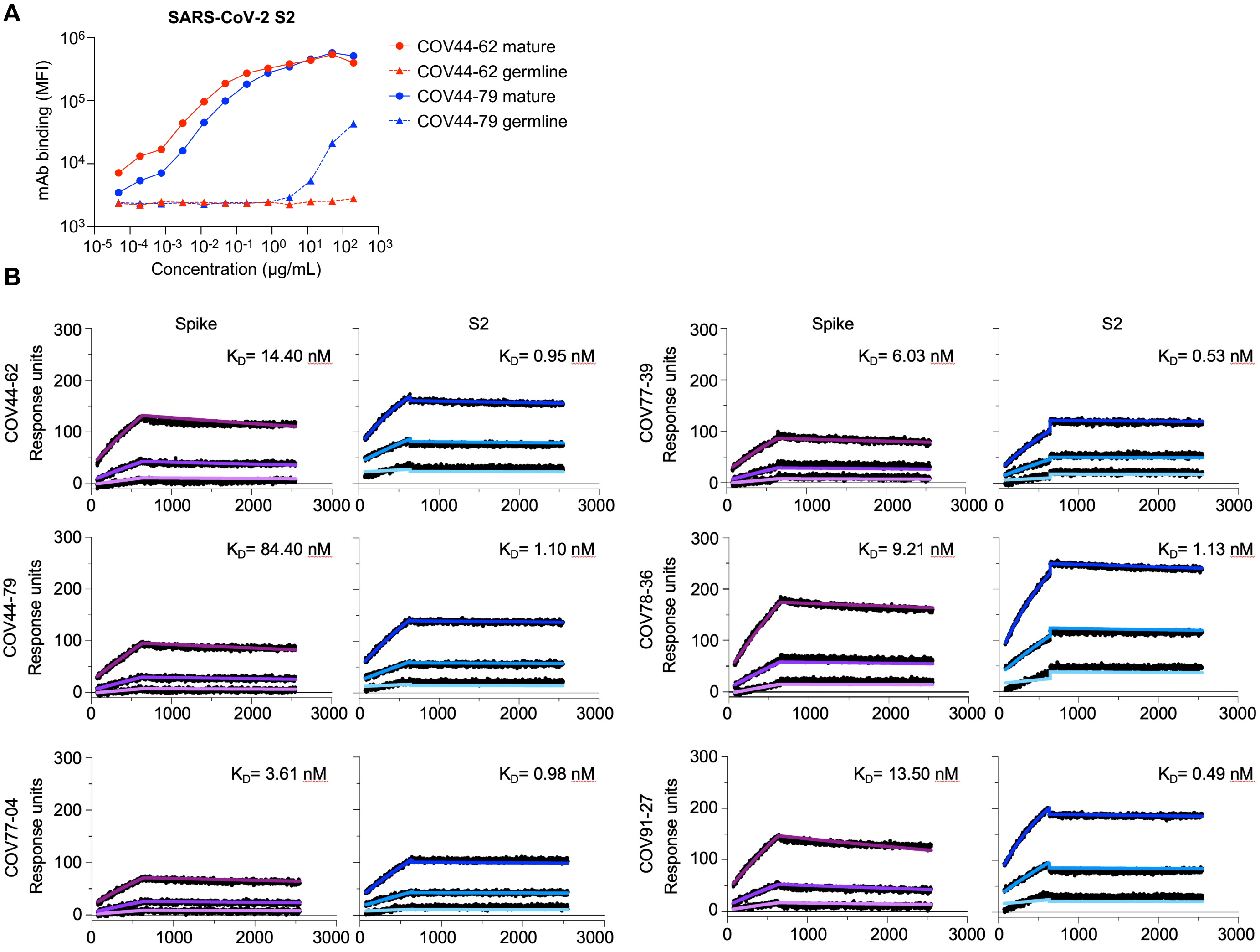

Fig. S2. Binding of fusion peptide mAbs to the S2 subunit. (**A**) Titration curves of mature and germline-reverted COV44-62 and COV44-79 binding to SARS-CoV-2 S2 in a bead-based assay. Interconnected data points are shown without curve fitting. (**B**) Kinetics curves for binding of fusion peptide Fabs to SARS-CoV-2 spike and S2 subunit. Curves are shown for binding to SARS-CoV-2 pre-fusion stabilized spike (2P) with an unmodified furin cleavage site and the non-pre-fusion stabilized S2 subunit. Black dots show raw data points and blue and purple lines show fitted curves.

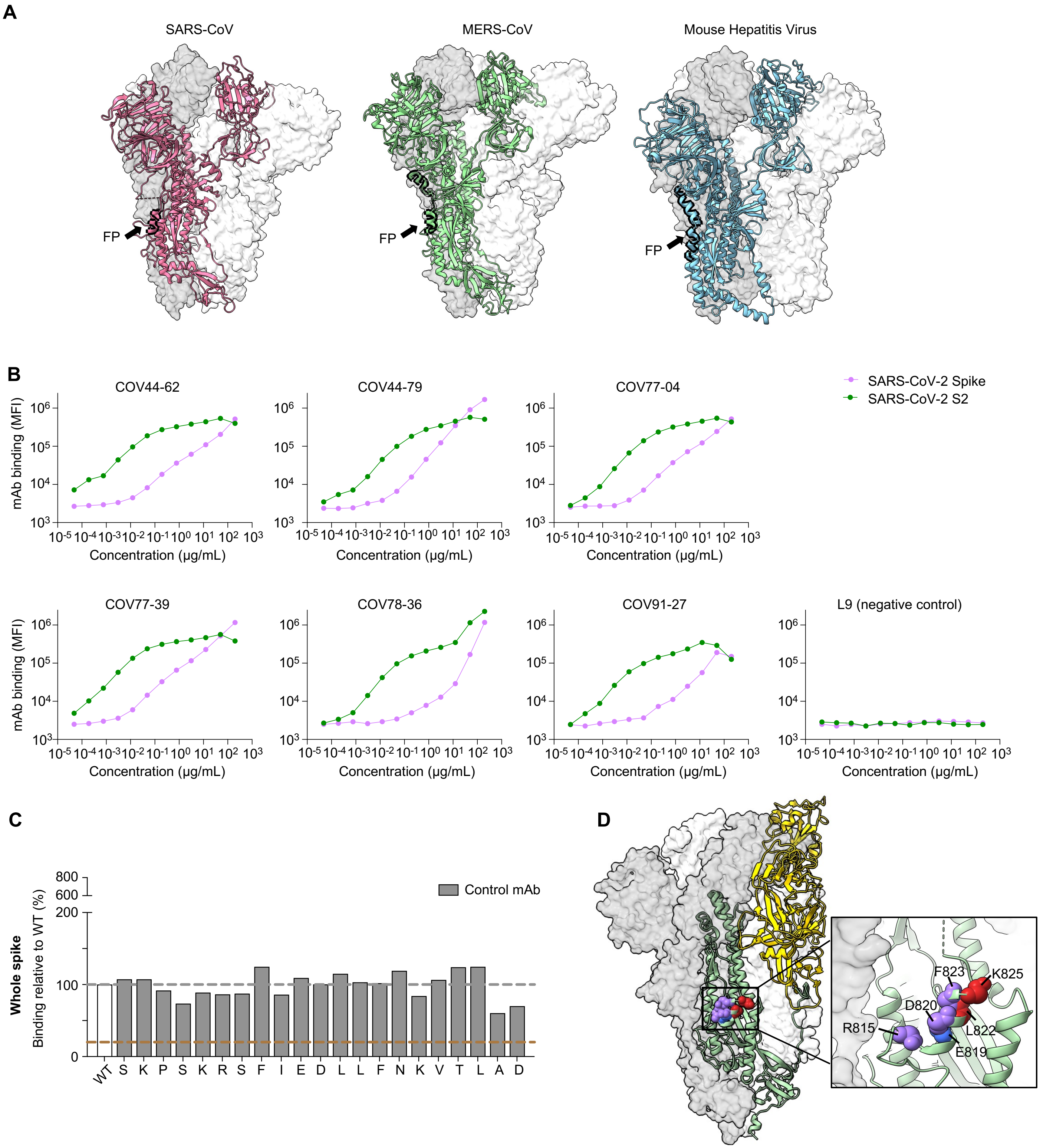

Fig. S3. Binding profile of broadly reactive mAbs targeting the fusion peptides. (**A**) Surface exposure of fusion peptide loops (highlighted in black) of SARS-CoV-1 (PDB ID 5X58), MERS-CoV-England 1 (PDB ID 6Q04), Mouse Hepatitis Virus (PDB ID 3JCL) amino-acid residues 870-897. The fusion peptide loop for each virus is highlighted in black corresponding to residues 870-897, 888-915, and 798-825, respectively. (**B**) Titration curves of broadly reactive mAb binding to SARS-CoV-2 spike trimer (2P, furin cleavage site intact) and unmodified SARS-CoV-2 S2 subunit monomer in a bead-based assay. Interconnected data points are shown without curve fitting. (**C**) Amino acids important for the binding of a negative control mAb identified by shotgun alanine mutagenesis of all residues on the S2 subunit. The control mAb binds to the spike protein but not to this region of the protein. Any residues where the binding of the control was <20% relative to wild-type spike was not considered a target residue of COV44-62, COV44-79 or COV77-39. Any alanines in the sequence were mutated to serines. (**D**) Residues important for the binding of COV44-62 and COV44-79, based on shotgun mutagenesis, are shown on the SARS-CoV-2 spike structure. Residues important for COV44-62 binding are in red, those important for COV44-79 are in blue, and those important for both are in purple. D830, which was identified as a target residue of COV44-62, was not resolved in this structure (PDB 6VSB) and is not shown here.

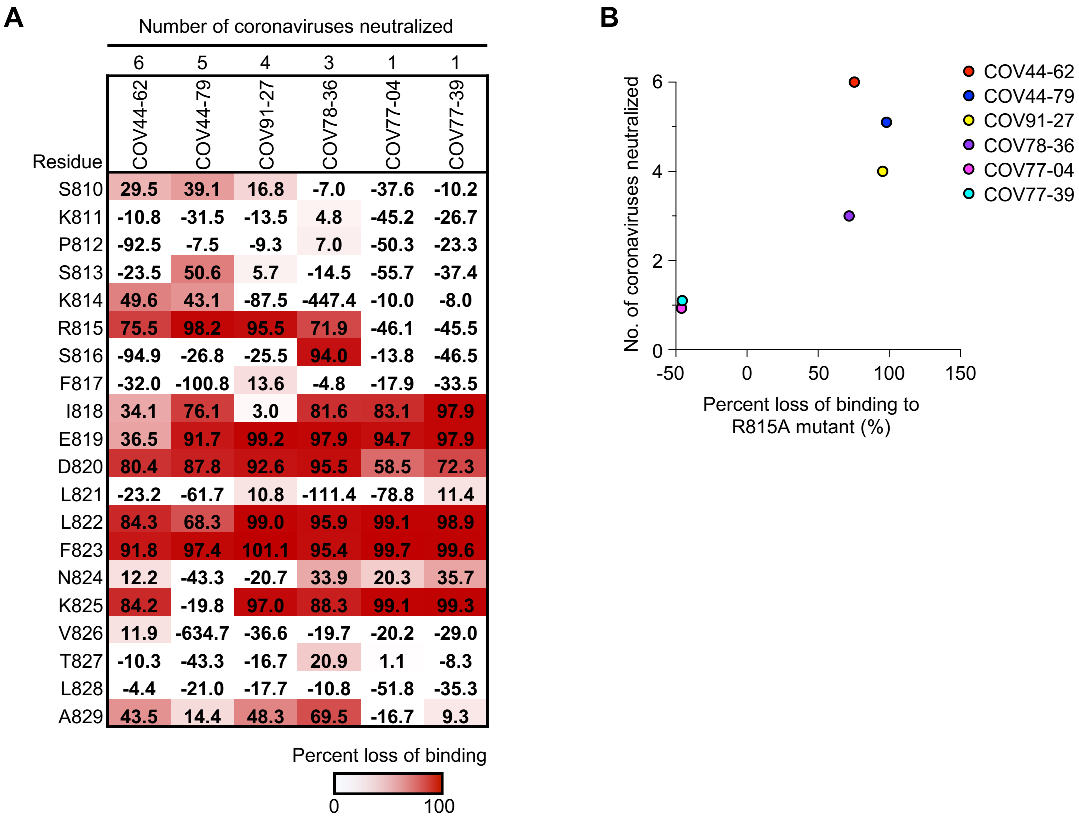

Fig. S4. Alanine scan shotgun mutagenesis of spike protein to identify important residues for binding of fusion peptide-specific mAbs. (**A**) Heat map showing percent loss of mAb binding to spike with a single mutation as indicated relative to wild-type spike protein. All mutations were to alanine except A829, which was mutated to serine. (**B**) Number of coronaviruses neutralized versus percentage loss of binding to the R815A mutant, relative to wild-type spike.

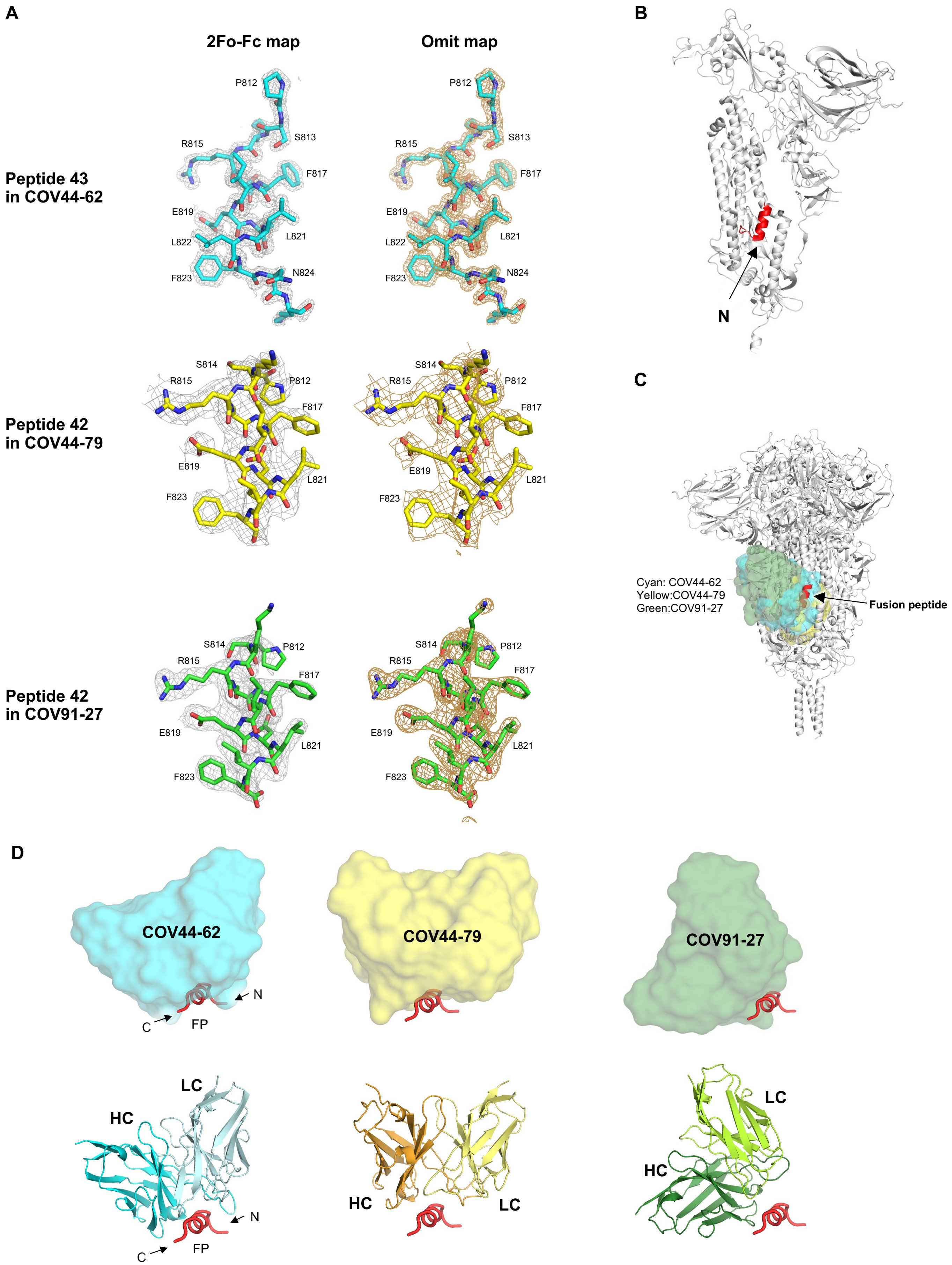
A
2Fo-Fc map
Omit map
Peptide 43
in COV44-62
Peptide 42
in COV44-79
Peptide 42
in COV91-27
P812
S813
R815
F817
E819
L821
L822
F823
N824
S814
B
N
C
Cyan: COV44-62
Yellow:COV44-79
Green:COV91-27
Fusion peptide
D
COV44-62
COV44-79
COV91-27
N
C
FP
LC
HC

Fig. S5. Electron density maps for the fusion peptides and binding angle of COV44-62, COV44-79, and COV91-27 to the fusion peptide. (**A**) The 2Fo-Fc electron density maps are represented in a gray mesh and contoured at 1.5σ, 0.6σ, and 0.8σ for the fusion peptides (FPs) bound to COV44-62, COV44-79, and COV91-27, respectively. The Fo-Fc unbiased omit electron density maps of the FPs are represented in a brown mesh and contoured at 3.0σ, 1.2σ, and 2.0σ, respectively. (**B**) Location of fusion peptide (in red) on a protomer of the SARS-CoV-2 spike protein (in white). (**C**) Structures of fusion peptides bound with antibodies COV44-62, COV44-79, and COV91-27 were superimposed onto the fusion peptide region (red) of an intact SARS-CoV-2 spike trimer structure in the pre-fusion state (PDB: 6XR8) (*69*). For clarity, only variable domains of the antibodies are shown. The antibodies clash with the spike protein when docked onto their fusion peptide epitopes. Conformational changes or conformational dynamics would be required to fully access the fusion peptide epitope. (**D**) Differences in binding angles of COV44-62, COV44-79, and COV91-27 on interaction with the fusion peptide. The antibodies are aligned on the fusion peptide. The fusion peptides are shown in the same orientation. For clarity, only the variable domains of the antibodies are shown as a molecular surface (top) and in ribbon representation (below).

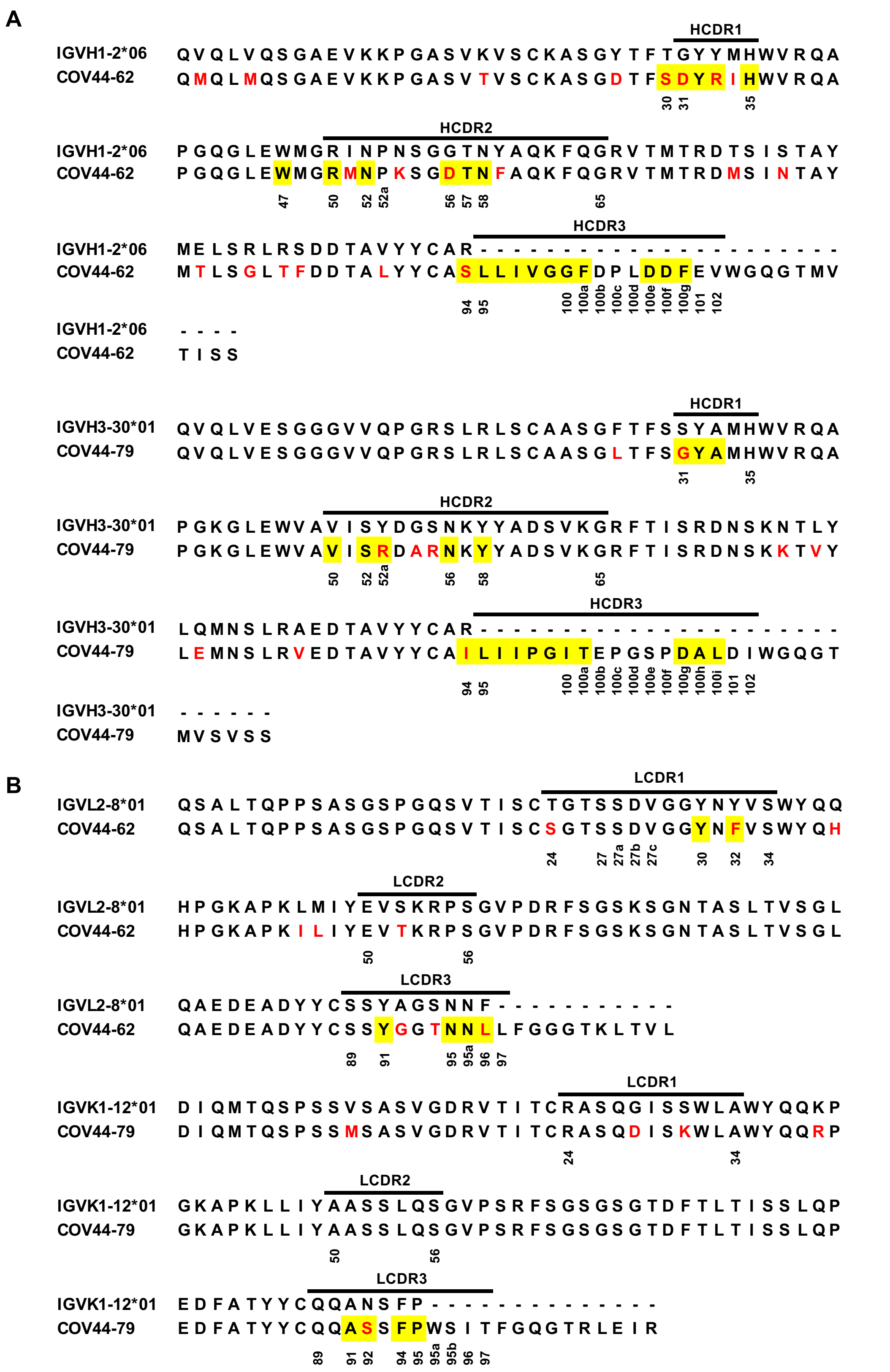

Fig. S6. Comparison of COV44-62 and COV44-79 sequences to the VH and VL germlines.
(**A**) Alignment of the heavy-chain variable domain sequences of COV44-62 and COV44-79 with the human germline IGVH1-2*06 and IGVH3-30*01 sequences, respectively.
(**B**) Alignment of the light-chain variable domain sequences of COV44-62 and COV44-79 with the human germline IGVL2-8*01 and IGVK1-12*01 sequences, respectively. Sequences that correspond to HCDR1, HCDR2, HCDR3, LCDR1, LCDR2, and LCDR3 are indicated. Residues that differ from the germline are in red. Residues interacting with the fusion peptide are highlighted in yellow. Residue are labeled in Kabat numbering.

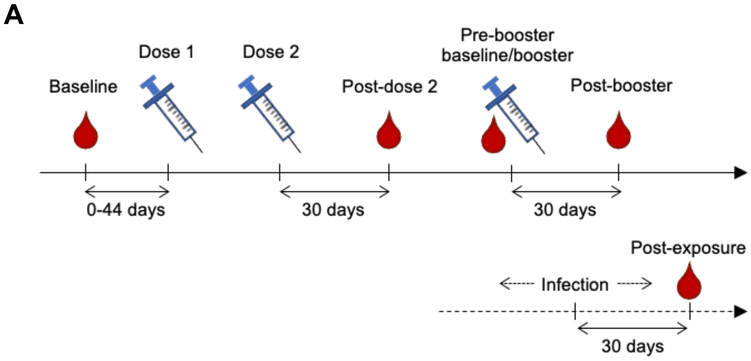

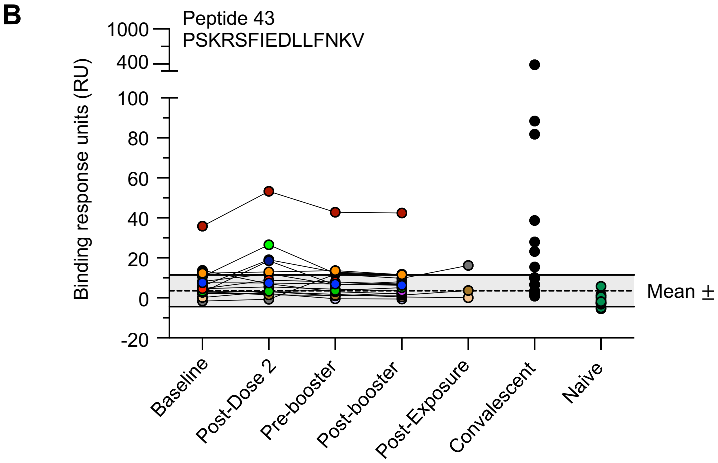

Analysis		*P-value*
Naive	Post-Dose 2	0.421
Naïve	Convalescent	**0.011**
Post-Dose 2	Baseline	**0.025**
Post-Dose 2	Pre-Booster	0.241
Post-Dose 2	Convalescent	0.864
Baseline	Post-Booster	1.000
Pre-Booster	Post-Booster	1.000

Fig. S7. Antibody responses to the fusion peptide after SARS-CoV-2 infection or mRNA-1273 vaccination. (**A**) Vaccination schedule showing plasma or serum collection time points. All individuals were vaccinated and boosted with the Moderna mRNA-1273 vaccine. For three out of 16 individuals, plasma was collected 30 days after documented infection with SARS-CoV-2. (**B**) Circulating IgG reactivity from vaccinated (n=16), convalescent unvaccinated (n=16) and COVID-19 naïve (n=13) individuals to peptide 43, a peptide covering the fusion peptide region just downstream of the S2' cleavage site. All polyclonal IgG was tested at 100 μg/mL. Background is represented as mean ± SD of the donors in the naïve and baseline groups. P values were computed from a nested, mixed-model ANOVA with Bonferroni-adjusted post hoc comparisons of the groups shown in the table. The high outlier in the convalescent group was excluded from the statistical analysis.

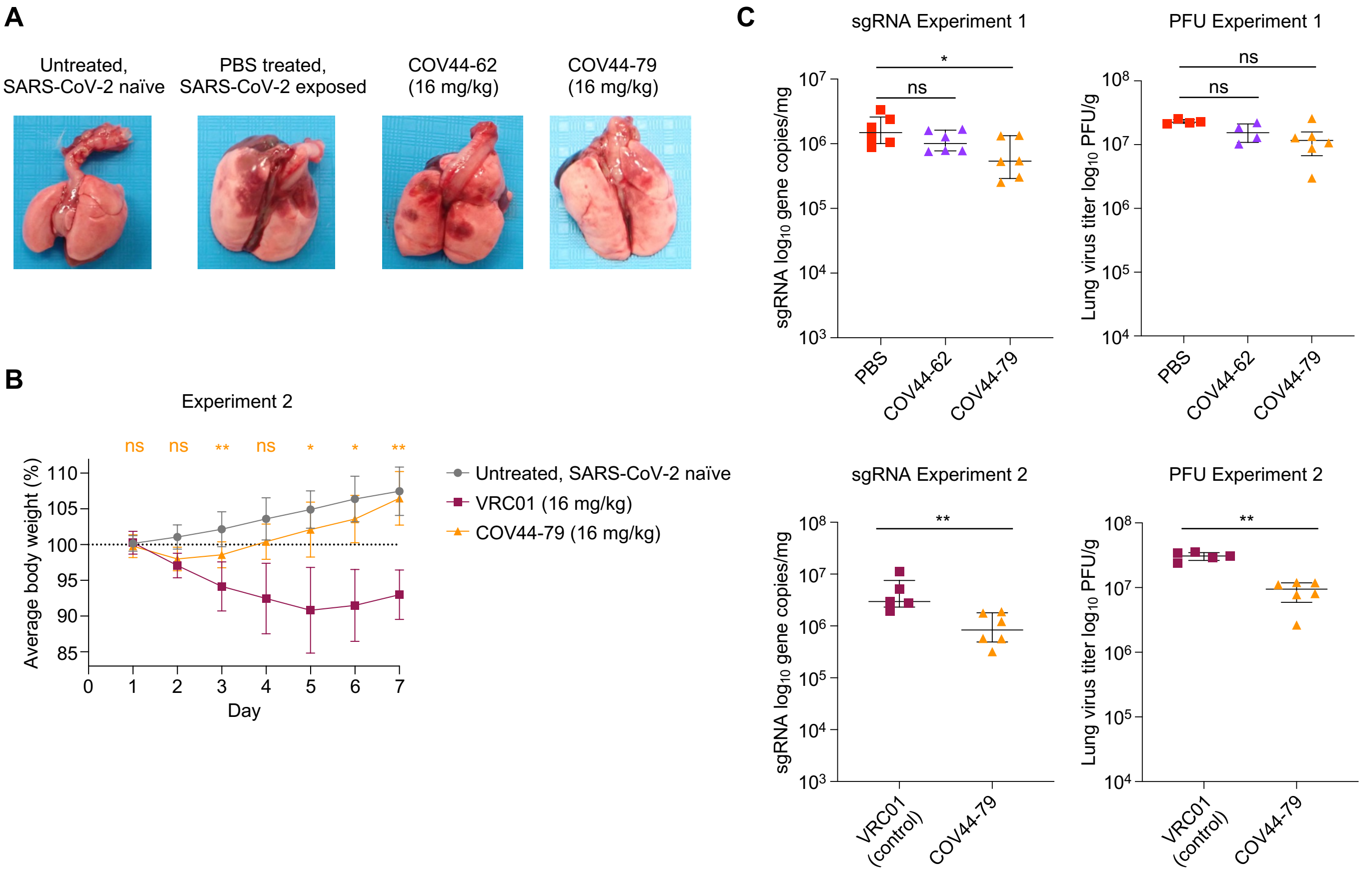

Fig. S8. COV44-79 limits disease in SARS-CoV-2 exposed Syrian hamsters. (**A**) Representative lung pathology of SARS-CoV-2 naïve and SARS-CoV-2 exposed Syrian hamsters from animals that received treatment with COV44-62, COV44-79 or PBS only (control). (**B**) Weight change for SARS-CoV-2 naïve animals versus virus-exposed animals that were mock-treated with hamster VRC01 or treated with COV44-79. Statistical significance for average body weight was analyzed across the 7-day time-course using a mixed-effects repeated measures model with Dunnett's post-test multiple comparison (n=9-12 animals from Day 0-3 and n=4-6 animals from Day 4-7). Error bars show mean $\pm$ SD. (**C**) Subgenomic RNA (sgRNA) levels in the lungs and lung virus titers measured based on a plaque forming assay. Samples were taken from hamsters on day 3 post-infection. Experiment 1 samples were analyzed by a Kruskal-Wallis test with Dunn's post-test multiple comparison (n=4-6 animals per condition), between the mAb-treated and mock-treated groups on each day. Experiment 2 samples were analyzed by a Mann-Whitney U-test. Bars show median + interquartile range. PFU, plaque forming units. $*P < 0.05$, $**P < 0.01$, $***P < 0.001$, $****P < 0.0001$ and ns, not significant.

Table S1. Isotype and V(D)J gene usage of fusion peptide-specific monoclonal antibodies.

mAb	Isotype	L	VH gene	% VH	DH gene	JH gene	%JH	HCDR3 length	HCDR3 sequence	VL gene	%VL	JL gene	%JL	LCDR3 length	LCDR3 sequence
COV44-62	IgG1	λ	VH1-2	88.5	DH3-10	JH3	92.0	17	ASLLIVGGFDPLDDFEV	VL2-8	96.2	JL3	97.1	10	SSYGGTNNLL
COV44-79	IgG1	κ	VH3-30 or VH3-30-3	95.8	DH6-19	JH3	96.0	19	AILIIPGITEPGSPDALDI	VK1-12 or VK1D-12	97.1	JK5	94.7	11	QQASSFPWSIT
COV77-04	IgG1	λ	VH4-59	90.2	DH2-8	JH4	87.5	11	ARTTTSVPFDY	VL3-16	91.8	JL3	94.4	10	LSTDGSTTWV
COV77-39	IgG1	λ	VH4-59	90.2	DH4-17	JH4	89.6	11	ARTTTTFPFDS	VL3-16	95.7	JL3	97.2	10	LSVDSTANWV
COV78-36	IgG1	λ	VH5-10-1	86.8	DH2-15	JH4	81.3	13	TTGAGVVIAASYF	VL2-8	94.1	JL2 or JL3	84.2	10	SSYSADSSII
COV91-27	IgG1	κ	VH3-30 or VH3-30-3	89.2	DH3-10	JH4	77.1	14	ATLGGWFEEASPTY	VK3-11	93.2	JK5	94.7	10	QQRGDWPLIT

Table S2. X-ray data collection and refinement statistics

**Data collection**	COV44-62 + SARS-CoV-2 FP	COV44-79 + SARS-CoV-2 FP	COV91-27 + SARS-CoV-2 FP
Beamline	SSRL12-1	APS 23-ID-B	SSRL12-1
Wavelength (Å)	0.9795	1.0337	0.9795
Space group	C 1 2 1	$P\ 4_1\ 2\ 2$	C 1 2 1
Unit cell parameters			
a, b, c (Å)	99.5, 71.9, 76.3	100.4, 100.4, 229.9	196.2, 60.3, 186.5
α, β, γ (°)	90, 114.2, 90	90, 90, 90	90, 109.5, 90
Resolution (Å) [a]	50.0-1.46 (1.49-1.46)	50.0-2.80 (2.85-2.80)	50.0-2.30 (2.34-2.30)
Unique reflections [a]	80,349 (5,949)	29,455 (2,761)	88,857 (8,409)
Redundancy [a]	5.6 (4.3)	10.5 (9.9)	4.8 (5.1)
Completeness (%) [a]	93.5 (65.0)	98.9 (95.9)	97.2 (98.5)
$<I/\sigma_I>$ [a]	31.2 (1.3)	8.0 (0.7)	13.0 (0.7)
R_{sym}[b] (%) [a]	6.9 (74.3)	17.9 (>100)	12.8 (>100)
R_{pim}[b] (%) [a]	3.1 (36.0)	5.6 (43.9)	6.7 (70.0)
$CC_{1/2}$[c] (%) [a]	99.6 (76.7)	98.6 (40.6)	99.0 (48.2)
**Refinement statistics**			
Resolution (Å)	36.0-1.46	46.0-2.80	46.1-2.30
Reflections (work)	80,321	29,422	88,514
Reflections (test)	4,003	1,454	4,453
R_{cryst}[d] / R_{free}[e] (%)	19.6/21.8	22.9/28.1	23.6/28.0
No. of copies in ASU	1	2	4
No. of atoms	3,877	6,918	13,790
Fab	3,230	6,714	13,209
Fusion peptide	122	204	398
Solvent	525	0	183
Average *B*-values (Å^2)	27	66	58
Fab	26	65	57
Fusion peptide	21	100	66
Solvent	38	N/A	57
Wilson *B*-value (Å^2)	17	64	58
**RMSD from ideal geometry**			
Bond length (Å)	0.007	0.002	0.005
Bond angle (°)	0.96	0.59	0.78
**Ramachandran statistics (%)** [f]			
Favored	97.2	95.3	97.3
Outliers	0.00	0.00	0.00
**PDB code**	8D36	8DAO	8D6Z

References and Notes

1. P. V'kovski, A. Kratzel, S. Steiner, H. Stalder, V. Thiel, Coronavirus biology and replication: Implications for SARS-CoV-2. *Nat. Rev. Microbiol.* **19**, 155–170 (2021). doi:10.1038/s41579-020-00468-6 Medline

2. E. Dong, H. Du, L. Gardner, An interactive web-based dashboard to track COVID-19 in real time. *Lancet Infect. Dis.* **20**, 533–534 (2020). doi:10.1016/S1473-3099(20)30120-1 Medline

3. S. Iketani, L. Liu, Y. Guo, L. Liu, J. F.-W. Chan, Y. Huang, M. Wang, Y. Luo, J. Yu, H. Chu, K. K.-H. Chik, T. T.-T. Yuen, M. T. Yin, M. E. Sobieszczyk, Y. Huang, K.-Y. Yuen, H. H. Wang, Z. Sheng, D. D. Ho, Antibody evasion properties of SARS-CoV-2 Omicron sublineages. *Nature* **604**, 553–556 (2022). doi:10.1038/s41586-022-04594-4 Medline

4. N. Andrews, J. Stowe, F. Kirsebom, S. Toffa, T. Rickeard, E. Gallagher, C. Gower, M. Kall, N. Groves, A.-M. O'Connell, D. Simons, P. B. Blomquist, A. Zaidi, S. Nash, N. Iwani Binti Abdul Aziz, S. Thelwall, G. Dabrera, R. Myers, G. Amirthalingam, S. Gharbia, J. C. Barrett, R. Elson, S. N. Ladhani, N. Ferguson, M. Zambon, C. N. J. Campbell, K. Brown, S. Hopkins, M. Chand, M. Ramsay, J. Lopez Bernal, Covid-19 vaccine effectiveness against the Omicron (B.1.1.529) variant. *N. Engl. J. Med.* **386**, 1532–1546 (2022). doi:10.1056/NEJMoa2119451 Medline

5. E. Takashita, N. Kinoshita, S. Yamayoshi, Y. Sakai-Tagawa, S. Fujisaki, M. Ito, K. Iwatsuki-Horimoto, S. Chiba, P. Halfmann, H. Nagai, M. Saito, E. Adachi, D. Sullivan, A. Pekosz, S. Watanabe, K. Maeda, M. Imai, H. Yotsuyanagi, H. Mitsuya, N. Ohmagari, M. Takeda, H. Hasegawa, Y. Kawaoka, Efficacy of antibodies and antiviral drugs against Covid-19 Omicron variant. *N. Engl. J. Med.* **386**, 995–998 (2022). doi:10.1056/NEJMc2119407 Medline

6. L. A. VanBlargan, J. M. Errico, P. J. Halfmann, S. J. Zost, J. E. Crowe Jr., L. A. Purcell, Y. Kawaoka, D. Corti, D. H. Fremont, M. S. Diamond, An infectious SARS-CoV-2 B.1.1.529 Omicron virus escapes neutralization by therapeutic monoclonal antibodies. *Nat. Med.* **28**, 490–495 (2022). doi:10.1038/s41591-021-01678-y Medline

7. J. A. Lednicky, M. S. Tagliamonte, S. K. White, M. A. Elbadry, M. M. Alam, C. J. Stephenson, T. S. Bonny, J. C. Loeb, T. Telisma, S. Chavannes, D. A. Ostrov, C. Mavian, V. M. Beau De Rochars, M. Salemi, J. G. Morris Jr., Independent infections of porcine deltacoronavirus among Haitian children. *Nature* **600**, 133–137 (2021). doi:10.1038/s41586-021-04111-z Medline

8. A. N. Vlasova, A. Diaz, D. Damtie, L. Xiu, T.-H. Toh, J. S.-Y. Lee, L. J. Saif, G. C. Gray, Novel canine coronavirus isolated from a hospitalized patient with pneumonia in East Malaysia. *Clin. Infect. Dis.* **74**, 446–454 (2022). doi:10.1093/cid/ciab456 Medline

9. C. B. Jackson, M. Farzan, B. Chen, H. Choe, Mechanisms of SARS-CoV-2 entry into cells. *Nat. Rev. Mol. Cell Biol.* **23**, 3–20 (2022). doi:10.1038/s41580-021-00418-x Medline

10. J. S. Tregoning, K. E. Flight, S. L. Higham, Z. Wang, B. F. Pierce, Progress of the COVID-19 vaccine effort: Viruses, vaccines and variants versus efficacy, effectiveness and escape. *Nat. Rev. Immunol.* **21**, 626–636 (2021). doi:10.1038/s41577-021-00592-1 Medline

11. T. N. Starr, A. J. Greaney, S. K. Hilton, D. Ellis, K. H. D. Crawford, A. S. Dingens, M. J. Navarro, J. E. Bowen, M. A. Tortorici, A. C. Walls, N. P. King, D. Veesler, J. D. Bloom, Deep mutational scanning of SARS-CoV-2 receptor binding domain reveals constraints on folding and ACE2 binding. *Cell* **182**, 1295–1310.e20 (2020). doi:10.1016/j.cell.2020.08.012 Medline

12. D. Pinto, M. M. Sauer, N. Czudnochowski, J. S. Low, M. A. Tortorici, M. P. Housley, J. Noack, A. C. Walls, J. E. Bowen, B. Guarino, L. E. Rosen, J. di Iulio, J. Jerak, H. Kaiser, S. Islam, S. Jaconi, N. Sprugasci, K. Culap, R. Abdelnabi, C. Foo, L. Coelmont, I. Bartha, S. Bianchi, C. Silacci-Fregni, J. Bassi, R. Marzi, E. Vetti, A. Cassotta, A. Ceschi, P. Ferrari, P. E. Cippà, O. Giannini, S. Ceruti, C. Garzoni, A. Riva, F. Benigni, E. Cameroni, L. Piccoli, M. S. Pizzuto, M. Smithey, D. Hong, A. Telenti, F. A. Lempp, J. Neyts, C. Havenar-Daughton, A. Lanzavecchia, F. Sallusto, G. Snell, H. W. Virgin, M. Beltramello, D. Corti, D. Veesler, Broad betacoronavirus neutralization by a stem helix-specific human antibody. *Science* **373**, 1109–1116 (2021). doi:10.1126/science.abj3321 Medline

13. M. M. Sauer, M. A. Tortorici, Y.-J. Park, A. C. Walls, L. Homad, O. J. Acton, J. E. Bowen, C. Wang, X. Xiong, W. de van der Schueren, J. Quispe, B. G. Hoffstrom, B.-J. Bosch, A. T. McGuire, D. Veesler, Structural basis for broad coronavirus neutralization. *Nat. Struct. Mol. Biol.* **28**, 478–486 (2021). doi:10.1038/s41594-021-00596-4 Medline

14. C. Wang, R. van Haperen, J. Gutiérrez-Álvarez, W. Li, N. M. A. Okba, I. Albulescu, I. Widjaja, B. van Dieren, R. Fernandez-Delgado, I. Sola, D. L. Hurdiss, O. Daramola, F. Grosveld, F. J. M. van Kuppeveld, B. L. Haagmans, L. Enjuanes, D. Drabek, B.-J. Bosch, A conserved immunogenic and vulnerable site on the coronavirus spike protein delineated by cross-reactive monoclonal antibodies. *Nat. Commun.* **12**, 1715 (2021). doi:10.1038/s41467-021-21968-w Medline

15. P. Zhou, M. Yuan, G. Song, N. Beutler, N. Shaabani, D. Huang, W. T. He, X. Zhu, S. Callaghan, P. Yong, F. Anzanello, L. Peng, J. Ricketts, M. Parren, E. Garcia, S. A. Rawlings, D. M. Smith, D. Nemazee, J. R. Teijaro, T. F. Rogers, I. A. Wilson, D. R. Burton, R. Andrabi, A human antibody reveals a conserved site on beta-coronavirus spike proteins and confers protection against SARS-CoV-2 infection. *Sci. Transl. Med.* **14**, eabi9215 (2022). doi:10.1126/scitranslmed.abi9215 Medline

16. W. Li, Y. Chen, J. Prévost, I. Ullah, M. Lu, S. Y. Gong, A. Tauzin, R. Gasser, D. Vézina, S. P. Anand, G. Goyette, D. Chaterjee, S. Ding, W. D. Tolbert, M. W. Grunst, Y. Bo, S. Zhang, J. Richard, F. Zhou, R. K. Huang, L. Esser, A. Zeher, M. Côté, P. Kumar, J. Sodroski, D. Xia, P. D. Uchil, M. Pazgier, A. Finzi, W. Mothes, Structural basis and mode of action for two broadly neutralizing antibodies against SARS-CoV-2 emerging variants of concern. *Cell Rep.* **38**, 110210 (2022). doi:10.1016/j.celrep.2021.110210 Medline

17. F. Amanat, M. Thapa, T. Lei, S. M. S. Ahmed, D. C. Adelsberg, J. M. Carreño, S. Strohmeier, A. J. Schmitz, S. Zafar, J. Q. Zhou, W. Rijnink, H. Alshammary, N. Borcherding, A. G. Reiche, K. Srivastava, E. M. Sordillo, H. van Bakel, J. S. Turner, G. Bajic, V. Simon, A. H. Ellebedy, F. Krammer, Personalized Virology Initiative, SARS-CoV-2 mRNA vaccination induces functionally diverse antibodies to NTD, RBD, and S2. *Cell* **184**, 3936–3948.e10 (2021). doi:10.1016/j.cell.2021.06.005 Medline

18. K. A. Huang, T. K. Tan, T.-H. Chen, C.-G. Huang, R. Harvey, S. Hussain, C.-P. Chen, A. Harding, J. Gilbert-Jaramillo, X. Liu, M. Knight, L. Schimanski, S.-R. Shih, Y.-C. Lin, C.-Y. Cheng, S.-H. Cheng, Y.-C. Huang, T.-Y. Lin, J.-T. Jan, C. Ma, W. James, R. S. Daniels, J. W. McCauley, P. Rijal, A. R. Townsend, Breadth and function of antibody response to acute SARS-CoV-2 infection in humans. *PLOS Pathog.* **17**, e1009352 (2021). doi:10.1371/journal.ppat.1009352 Medline

19. W. N. Voss, Y. J. Hou, N. V. Johnson, G. Delidakis, J. E. Kim, K. Javanmardi, A. P. Horton, F. Bartzoka, C. J. Paresi, Y. Tanno, C.-W. Chou, S. A. Abbasi, W. Pickens, K. George, D. R. Boutz, D. M. Towers, J. R. McDaniel, D. Billick, J. Goike, L. Rowe, D. Batra, J. Pohl, J. Lee, S. Gangappa, S. Sambhara, M. Gadush, N. Wang, M. D. Person, B. L. Iverson, J. D. Gollihar, J. M. Dye, A. S. Herbert, I. J. Finkelstein, R. S. Baric, J. S. McLellan, G. Georgiou, J. J. Lavinder, G. C. Ippolito, Prevalent, protective, and convergent IgG recognition of SARS-CoV-2 non-RBD spike epitopes. *Science* **372**, 1108–1112 (2021). doi:10.1126/science.abg5268 Medline

20. H. Cho, K. K. Gonzales-Wartz, D. Huang, M. Yuan, M. Peterson, J. Liang, N. Beutler, J. L. Torres, Y. Cong, E. Postnikova, S. Bangaru, C. A. Talana, W. Shi, E. S. Yang, Y. Zhang, K. Leung, L. Wang, L. Peng, J. Skinner, S. Li, N. C. Wu, H. Liu, C. Dacon, T. Moyer, M. Cohen, M. Zhao, F. E.-H. Lee, R. S. Weinberg, I. Douagi, R. Gross, C. Schmaljohn, A. Pegu, J. R. Mascola, M. Holbrook, D. Nemazee, T. F. Rogers, A. B. Ward, I. A. Wilson, P. D. Crompton, J. Tan, Bispecific antibodies targeting distinct regions of the spike protein potently neutralize SARS-CoV-2 variants of concern. *Sci. Transl. Med.* **13**, eabj5413 (2021). doi:10.1126/scitranslmed.abj5413 Medline

21. A. C. Walls, M. A. Tortorici, B.-J. Bosch, B. Frenz, P. J. M. Rottier, F. DiMaio, F. A. Rey, D. Veesler, Cryo-electron microscopy structure of a coronavirus spike glycoprotein trimer. *Nature* **531**, 114–117 (2016). doi:10.1038/nature16988 Medline

22. Y. Yuan, D. Cao, Y. Zhang, J. Ma, J. Qi, Q. Wang, G. Lu, Y. Wu, J. Yan, Y. Shi, X. Zhang, G. F. Gao, Cryo-EM structures of MERS-CoV and SARS-CoV spike glycoproteins reveal the dynamic receptor binding domains. *Nat. Commun.* **8**, 15092 (2017). doi:10.1038/ncomms15092 Medline

23. C. L. Hsieh, J. A. Goldsmith, J. M. Schaub, A. M. DiVenere, H.-C. Kuo, K. Javanmardi, K. C. Le, D. Wrapp, A. G. Lee, Y. Liu, C.-W. Chou, P. O. Byrne, C. K. Hjorth, N. V. Johnson, J. Ludes-Meyers, A. W. Nguyen, J. Park, N. Wang, D. Amengor, J. J. Lavinder, G. C. Ippolito, J. A. Maynard, I. J. Finkelstein, J. S. McLellan, Structure-based design of prefusion-stabilized SARS-CoV-2 spikes. *Science* **369**, 1501–1505 (2020). doi:10.1126/science.abd0826 Medline

24. J. F. Chan, A. J. Zhang, S. Yuan, V. K. Poon, C. C. Chan, A. C. Lee, W. M. Chan, Z. Fan, H. W. Tsoi, L. Wen, R. Liang, J. Cao, Y. Chen, K. Tang, C. Luo, J. P. Cai, K. H. Kok, H. Chu, K. H. Chan, S. Sridhar, Z. Chen, H. Chen, K. K. To, K. Y. Yuen, Simulation of the clinical and pathological manifestations of coronavirus disease 2019 (COVID-19) in a golden Syrian hamster model: Implications for disease pathogenesis and transmissibility. *Clin. Infect. Dis.* **71**, 2428–2446 (2020). doi:10.1093/cid/ciaa325 Medline

25. M. Imai, K. Iwatsuki-Horimoto, M. Hatta, S. Loeber, P. J. Halfmann, N. Nakajima, T. Watanabe, M. Ujie, K. Takahashi, M. Ito, S. Yamada, S. Fan, S. Chiba, M. Kuroda, L. Guan, K. Takada, T. Armbrust, A. Balogh, Y. Furusawa, M. Okuda, H. Ueki, A.

Yasuhara, Y. Sakai-Tagawa, T. J. S. Lopes, M. Kiso, S. Yamayoshi, N. Kinoshita, N. Ohmagari, S. I. Hattori, M. Takeda, H. Mitsuya, F. Krammer, T. Suzuki, Y. Kawaoka, Syrian hamsters as a small animal model for SARS-CoV-2 infection and countermeasure development. *Proc. Natl. Acad. Sci. U.S.A.* **117**, 16587–16595 (2020). doi:10.1073/pnas.2009799117 Medline

26. S. F. Sia, L.-M. Yan, A. W. H. Chin, K. Fung, K.-T. Choy, A. Y. L. Wong, P. Kaewpreedee, R. A. P. M. Perera, L. L. M. Poon, J. M. Nicholls, M. Peiris, H.-L. Yen, Pathogenesis and transmission of SARS-CoV-2 in golden hamsters. *Nature* **583**, 834–838 (2020). doi:10.1038/s41586-020-2342-5 Medline

27. R. Kong, K. Xu, T. Zhou, P. Acharya, T. Lemmin, K. Liu, G. Ozorowski, C. Soto, J. D. Taft, R. T. Bailer, E. M. Cale, L. Chen, C. W. Choi, G.-Y. Chuang, N. A. Doria-Rose, A. Druz, I. S. Georgiev, J. Gorman, J. Huang, M. G. Joyce, M. K. Louder, X. Ma, K. McKee, S. O'Dell, M. Pancera, Y. Yang, S. C. Blanchard, W. Mothes, D. R. Burton, W. C. Koff, M. Connors, A. B. Ward, P. D. Kwong, J. R. Mascola, Fusion peptide of HIV-1 as a site of vulnerability to neutralizing antibody. *Science* **352**, 828–833 (2016). doi:10.1126/science.aae0474 Medline

28. K. Xu, P. Acharya, R. Kong, C. Cheng, G.-Y. Chuang, K. Liu, M. K. Louder, S. O'Dell, R. Rawi, M. Sastry, C.-H. Shen, B. Zhang, T. Zhou, M. Asokan, R. T. Bailer, M. Chambers, X. Chen, C. W. Choi, V. P. Dandey, N. A. Doria-Rose, A. Druz, E. T. Eng, S. K. Farney, K. E. Foulds, H. Geng, I. S. Georgiev, J. Gorman, K. R. Hill, A. J. Jafari, Y. D. Kwon, Y.-T. Lai, T. Lemmin, K. McKee, T. Y. Ohr, L. Ou, D. Peng, A. P. Rowshan, Z. Sheng, J.-P. Todd, Y. Tsybovsky, E. G. Viox, Y. Wang, H. Wei, Y. Yang, A. F. Zhou, R. Chen, L. Yang, D. G. Scorpio, A. B. McDermott, L. Shapiro, B. Carragher, C. S. Potter, J. R. Mascola, P. D. Kwong, Epitope-based vaccine design yields fusion peptide-directed antibodies that neutralize diverse strains of HIV-1. *Nat. Med.* **24**, 857–867 (2018). doi:10.1038/s41591-018-0042-6 Medline

29. R. Kong, H. Duan, Z. Sheng, K. Xu, P. Acharya, X. Chen, C. Cheng, A. S. Dingens, J. Gorman, M. Sastry, C.-H. Shen, B. Zhang, T. Zhou, G.-Y. Chuang, C. W. Chao, Y. Gu, A. J. Jafari, M. K. Louder, S. O'Dell, A. P. Rowshan, E. G. Viox, Y. Wang, C. W. Choi, M. M. Corcoran, A. R. Corrigan, V. P. Dandey, E. T. Eng, H. Geng, K. E. Foulds, Y. Guo, Y. D. Kwon, B. Lin, K. Liu, R. D. Mason, M. C. Nason, T. Y. Ohr, L. Ou, R. Rawi, E. K. Sarfo, A. Schön, J. P. Todd, S. Wang, H. Wei, W. Wu, J. C. Mullikin, R. T. Bailer, N. A. Doria-Rose, G. B. Karlsson Hedestam, D. G. Scorpio, J. Overbaugh, J. D. Bloom, B. Carragher, C. S. Potter, L. Shapiro, P. D. Kwong, J. R. Mascola; NISC Comparative Sequencing Program, Antibody lineages with vaccine-induced antigen-binding hotspots develop broad HIV neutralization. *Cell* **178**, 567–584.e19 (2019). doi:10.1016/j.cell.2019.06.030 Medline

30. M. A. Tortorici, N. Czudnochowski, T. N. Starr, R. Marzi, A. C. Walls, F. Zatta, J. E. Bowen, S. Jaconi, J. Di Iulio, Z. Wang, A. De Marco, S. K. Zepeda, D. Pinto, Z. Liu, M. Beltramello, I. Bartha, M. P. Housley, F. A. Lempp, L. E. Rosen, E. Dellota Jr., H. Kaiser, M. Montiel-Ruiz, J. Zhou, A. Addetia, B. Guarino, K. Culap, N. Sprugasci, C. Saliba, E. Vetti, I. Giacchetto-Sasselli, C. S. Fregni, R. Abdelnabi, S. C. Foo, C. Havenar-Daughton, M. A. Schmid, F. Benigni, E. Cameroni, J. Neyts, A. Telenti, H. W. Virgin, S. P. J. Whelan, G. Snell, J. D. Bloom, D. Corti, D. Veesler, M. S. Pizzuto, Broad

sarbecovirus neutralization by a human monoclonal antibody. *Nature* **597**, 103–108 (2021). doi:10.1038/s41586-021-03817-4 Medline

31. I. Ullah, J. Prévost, M. S. Ladinsky, H. Stone, M. Lu, S. P. Anand, G. Beaudoin-Bussières, K. Symmes, M. Benlarbi, S. Ding, R. Gasser, C. Fink, Y. Chen, A. Tauzin, G. Goyette, C. Bourassa, H. Medjahed, M. Mack, K. Chung, C. B. Wilen, G. A. Dekaban, J. D. Dikeakos, E. A. Bruce, D. E. Kaufmann, L. Stamatatos, A. T. McGuire, J. Richard, M. Pazgier, P. J. Bjorkman, W. Mothes, A. Finzi, P. Kumar, P. D. Uchil, Live imaging of SARS-CoV-2 infection in mice reveals that neutralizing antibodies require Fc function for optimal efficacy. *Immunity* **54**, 2143–2158.e15 (2021). doi:10.1016/j.immuni.2021.08.015 Medline

32. R. Yamin, A. T. Jones, H.-H. Hoffmann, A. Schäfer, K. S. Kao, R. L. Francis, T. P. Sheahan, R. S. Baric, C. M. Rice, J. V. Ravetch, S. Bournazos, Fc-engineered antibody therapeutics with improved anti-SARS-CoV-2 efficacy. *Nature* **599**, 465–470 (2021). doi:10.1038/s41586-021-04017-w Medline

33. Y. C. Bartsch, C. Wang, T. Zohar, S. Fischinger, C. Atyeo, J. S. Burke, J. Kang, A. G. Edlow, A. Fasano, L. R. Baden, E. J. Nilles, A. E. Woolley, E. W. Karlson, A. R. Hopke, D. Irimia, E. S. Fischer, E. T. Ryan, R. C. Charles, B. D. Julg, D. A. Lauffenburger, L. M. Yonker, G. Alter, Humoral signatures of protective and pathological SARS-CoV-2 infection in children. *Nat. Med.* **27**, 454–462 (2021). doi:10.1038/s41591-021-01263-3 Medline

34. C. H. Shen, B. J. DeKosky, Y. Guo, K. Xu, Y. Gu, D. Kilam, S. H. Ko, R. Kong, K. Liu, M. K. Louder, L. Ou, B. Zhang, C. W. Chao, M. M. Corcoran, E. Feng, J. Huang, E. Normandin, S. O'Dell, A. Ransier, R. Rawi, M. Sastry, S. D. Schmidt, S. Wang, Y. Wang, G.-Y. Chuang, N. A. Doria-Rose, B. Lin, T. Zhou, E. A. Boritz, M. Connors, D. C. Douek, G. B. Karlsson Hedestam, Z. Sheng, L. Shapiro, J. R. Mascola, P. D. Kwong, VRC34-antibody lineage development reveals how a required rare mutation shapes the maturation of a broad HIV-neutralizing lineage. *Cell Host Microbe* **27**, 531–543.e6 (2020). doi:10.1016/j.chom.2020.01.027 Medline

35. S. Kratochvil, C.-H. Shen, Y.-C. Lin, K. Xu, U. Nair, L. Da Silva Pereira, P. Tripathi, J. Arnold, G.-Y. Chuang, E. Melzi, A. Schön, B. Zhang, M. Dillon, B. Bonilla, B. J. Flynn, K. H. Kirsch, N. K. Kisalu, P. K. Kiyuka, T. Liu, L. Ou, M. Pancera, R. Rawi, M. Reveiz, K. Seignon, L. T. Wang, M. T. Waring, J. Warner, Y. Yang, J. R. Francica, A. H. Idris, R. A. Seder, P. D. Kwong, F. D. Batista, Vaccination in a humanized mouse model elicits highly protective PfCSP-targeting anti-malarial antibodies. *Immunity* **54**, 2859–2876.e7 (2021). doi:10.1016/j.immuni.2021.10.017 Medline

36. A. Wellner, C. McMahon, M. S. A. Gilman, J. R. Clements, S. Clark, K. M. Nguyen, M. H. Ho, V. J. Hu, J.-E. Shin, J. Feldman, B. M. Hauser, T. M. Caradonna, L. M. Wingler, A. G. Schmidt, D. S. Marks, J. Abraham, A. C. Kruse, C. C. Liu, Rapid generation of potent antibodies by autonomous hypermutation in yeast. *Nat. Chem. Biol.* **17**, 1057–1064 (2021). doi:10.1038/s41589-021-00832-4 Medline

37. C. M. Poh, G. Carissimo, B. Wang, S. N. Amrun, C. Y.-P. Lee, R. S.-L. Chee, S.-W. Fong, N. K.-W. Yeo, W.-H. Lee, A. Torres-Ruesta, Y.-S. Leo, M. I.-C. Chen, S.-Y. Tan, L. Y. A. Chai, S. Kalimuddin, S. S. G. Kheng, S.-Y. Thien, B. E. Young, D. C. Lye, B. J. Hanson, C.-I. Wang, L. Renia, L. F. P. Ng, Two linear epitopes on the SARS-CoV-2

spike protein that elicit neutralising antibodies in COVID-19 patients. *Nat. Commun.* **11**, 2806 (2020). doi:10.1038/s41467-020-16638-2 Medline

38. W. R. Morgenlander, S. N. Henson, D. R. Monaco, A. Chen, K. Littlefield, E. M. Bloch, E. Fujimura, I. Ruczinski, A. R. Crowley, H. Natarajan, S. E. Butler, J. A. Weiner, M. Z. Li, T. S. Bonny, S. E. Benner, A. Balagopal, D. Sullivan, S. Shoham, T. C. Quinn, S. H. Eshleman, A. Casadevall, A. D. Redd, O. Laeyendecker, M. E. Ackerman, A. Pekosz, S. J. Elledge, M. Robinson, A. A. R. Tobian, H. B. Larman, Antibody responses to endemic coronaviruses modulate COVID-19 convalescent plasma functionality. *J. Clin. Invest.* **131**, e146927 (2021). doi:10.1172/JCI146927 Medline

39. X. Sun *et al.*, Novel neutralization mechanism of a human antibody with pan-coronavirus reactivity. *Res Sq*, [Preprint] (2021); https://doi.org/10.21203/rs.3.rs-952553/v1.

40. N. Vanderheijden, A. Stevaert, J. Xie, X. Ren, C. Barbezange, S. Noppen, I. Desombere, B. Verhasselt, P. Geldhof, N. Vereecke, V. Stroobants, D. Oh, M. Vanhee, L. M. J. Naesens, H. J. Nauwynck, Functional analysis of human and feline coronavirus cross-reactive antibodies directed against the SARS-CoV-2 fusion peptide. *Front. Immunol.* **12**, 790415 (2022). doi:10.3389/fimmu.2021.790415 Medline

41. C. Dreyfus, N. S. Laursen, T. Kwaks, D. Zuijdgeest, R. Khayat, D. C. Ekiert, J. H. Lee, Z. Metlagel, M. V. Bujny, M. Jongeneelen, R. van der Vlugt, M. Lamrani, H. J. W. M. Korse, E. Geelen, Ö. Sahin, M. Sieuwerts, J. P. J. Brakenhoff, R. Vogels, O. T. W. Li, L. L. M. Poon, M. Peiris, W. Koudstaal, A. B. Ward, I. A. Wilson, J. Goudsmit, R. H. E. Friesen, Highly conserved protective epitopes on influenza B viruses. *Science* **337**, 1343–1348 (2012). doi:10.1126/science.1222908 Medline

42. T. F. Rogers, F. Zhao, D. Huang, N. Beutler, A. Burns, W. T. He, O. Limbo, C. Smith, G. Song, J. Woehl, L. Yang, R. K. Abbott, S. Callaghan, E. Garcia, J. Hurtado, M. Parren, L. Peng, S. Ramirez, J. Ricketts, M. J. Ricciardi, S. A. Rawlings, N. C. Wu, M. Yuan, D. M. Smith, D. Nemazee, J. R. Teijaro, J. E. Voss, I. A. Wilson, R. Andrabi, B. Briney, E. Landais, D. Sok, J. G. Jardine, D. R. Burton, Isolation of potent SARS-CoV-2 neutralizing antibodies and protection from disease in a small animal model. *Science* **369**, 956–963 (2020). doi:10.1126/science.abc7520 Medline

43. L. T. Wang, L. S. Pereira, Y. Flores-Garcia, J. O'Connor, B. J. Flynn, A. Schön, N. K. Hurlburt, M. Dillon, A. S. P. Yang, A. Fabra-García, A. H. Idris, B. T. Mayer, M. W. Gerber, R. Gottardo, R. D. Mason, N. Cavett, R. B. Ballard, N. K. Kisalu, A. Molina-Cruz, J. Nelson, R. Vistein, C. Barillas-Mury, R. Amino, D. Baker, N. P. King, R. W. Sauerwein, M. Pancera, I. A. Cockburn, F. Zavala, J. R. Francica, R. A. Seder, A potent anti-malarial human monoclonal antibody targets circumsporozoite protein minor repeats and neutralizes sporozoites in the liver. *Immunity* **53**, 733–744.e8 (2020). doi:10.1016/j.immuni.2020.08.014 Medline

44. E. Krissinel, K. Henrick, Inference of macromolecular assemblies from crystalline state. *J. Mol. Biol.* **372**, 774–797 (2007). doi:10.1016/j.jmb.2007.05.022 Medline

45. S. Bangaru, A. Antanasijevic, N. Kose, L. M. Sewall, A. M. Jackson, N. Suryadevara, X. Zhan, J. L. Torres, J. Copps, A. T. de la Peña, J. E. Crowe Jr., A. B. Ward, Structural mapping of antibody landscapes to human betacoronavirus spike proteins. *Sci. Adv.* **8**, eabn2911 (2022). doi:10.1126/sciadv.abn2911 Medline

46. H. Lv, O. T.-Y. Tsang, R. T. Y. So, Y. Wang, M. Yuan, H. Liu, G. K. Yip, Q. W. Teo, Y. Lin, W. Liang, J. Wang, W. W. Ng, I. A. Wilson, J. S. M. Peiris, N. C. Wu, C. K. P. Mok, Homologous and heterologous serological response to the N-terminal domain of SARS-CoV-2 in humans and mice. *Eur. J. Immunol.* **51**, 2296–2305 (2021). doi:10.1002/eji.202149234 Medline

47. M. Yuan, N. C. Wu, X. Zhu, C. D. Lee, R. T. Y. So, H. Lv, C. K. P. Mok, I. A. Wilson, A highly conserved cryptic epitope in the receptor binding domains of SARS-CoV-2 and SARS-CoV. *Science* **368**, 630–633 (2020). doi:10.1126/science.abb7269 Medline

48. D. C. Ekiert, G. Bhabha, M.-A. Elsliger, R. H. E. Friesen, M. Jongeneelen, M. Throsby, J. Goudsmit, I. A. Wilson, Antibody recognition of a highly conserved influenza virus epitope. *Science* **324**, 246–251 (2009). doi:10.1126/science.1171491 Medline

49. C. Crosnier, M. Wanaguru, B. McDade, F. H. Osier, K. Marsh, J. C. Rayner, G. J. Wright, A library of functional recombinant cell-surface and secreted *P. falciparum* merozoite proteins. *Mol. Cell. Proteomics* **12**, 3976–3986 (2013). doi:10.1074/mcp.O113.028357 Medline

50. J. Huang, N. A. Doria-Rose, N. S. Longo, L. Laub, C.-L. Lin, E. Turk, B. H. Kang, S. A. Migueles, R. T. Bailer, J. R. Mascola, M. Connors, Isolation of human monoclonal antibodies from peripheral blood B cells. *Nat. Protoc.* **8**, 1907–1915 (2013). doi:10.1038/nprot.2013.117 Medline

51. S. Moir, R. Lapointe, A. Malaspina, M. Ostrowski, C. E. Cole, T.-W. Chun, J. Adelsberger, M. Baseler, P. Hwu, A. S. Fauci, CD40-Mediated induction of CD4 and CXCR4 on B lymphocytes correlates with restricted susceptibility to human immunodeficiency virus type 1 infection: Potential role of B lymphocytes as a viral reservoir. *J. Virol.* **73**, 7972–7980 (1999). doi:10.1128/JVI.73.10.7972-7980.1999 Medline

52. T. Tiller, E. Meffre, S. Yurasov, M. Tsuiji, M. C. Nussenzweig, H. Wardemann, Efficient generation of monoclonal antibodies from single human B cells by single cell RT-PCR and expression vector cloning. *J. Immunol. Methods* **329**, 112–124 (2008). doi:10.1016/j.jim.2007.09.017 Medline

53. M. P. Lefranc, Immunoglobulin and T cell receptor genes: IMGT® and the birth and rise of immunoinformatics. *Front. Immunol.* **5**, 22 (2014). doi:10.3389/fimmu.2014.00022 Medline

54. K. Katoh, J. Rozewicki, K. D. Yamada, MAFFT online service: Multiple sequence alignment, interactive sequence choice and visualization. *Brief. Bioinform.* **20**, 1160–1166 (2019). doi:10.1093/bib/bbx108 Medline

55. I. Letunic, P. Bork, Interactive Tree Of Life (iTOL) v5: An online tool for phylogenetic tree display and annotation. *Nucleic Acids Res.* **49**, W293–W296 (2021). doi:10.1093/nar/gkab301 Medline

56. G. E. Crooks, G. Hon, J. M. Chandonia, S. E. Brenner, WebLogo: A sequence logo generator. *Genome Res.* **14**, 1188–1190 (2004). doi:10.1101/gr.849004 Medline

57. J. Schindelin, I. Arganda-Carreras, E. Frise, V. Kaynig, M. Longair, T. Pietzsch, S. Preibisch, C. Rueden, S. Saalfeld, B. Schmid, J.-Y. Tinevez, D. J. White, V. Hartenstein, K. Eliceiri, P. Tomancak, A. Cardona, Fiji: An open-source platform for biological-image analysis. *Nat. Methods* **9**, 676–682 (2012). doi:10.1038/nmeth.2019 Medline

58. L. Lu, Q. Liu, Y. Zhu, K.-H. Chan, L. Qin, Y. Li, Q. Wang, J. F.-W. Chan, L. Du, F. Yu, C. Ma, S. Ye, K.-Y. Yuen, R. Zhang, S. Jiang, Structure-based discovery of Middle East respiratory syndrome coronavirus fusion inhibitor. *Nat. Commun.* **5**, 3067 (2014). doi:10.1038/ncomms4067 Medline

59. E. Davidson, B. J. Doranz, A high-throughput shotgun mutagenesis approach to mapping B-cell antibody epitopes. *Immunology* **143**, 13–20 (2014). doi:10.1111/imm.12323 Medline

60. Z. Otwinowski, W. Minor, Processing of X-ray diffraction data collected in oscillation mode. *Methods Enzymol.* **276**, 307–326 (1997). doi:10.1016/S0076-6879(97)76066-X Medline

61. A. J. McCoy, R. W. Grosse-Kunstleve, P. D. Adams, M. D. Winn, L. C. Storoni, R. J. Read, Phaser crystallographic software. *J. Appl. Crystallogr.* **40**, 658–674 (2007). doi:10.1107/S0021889807021206 Medline

62. P. Emsley, B. Lohkamp, W. G. Scott, K. Cowtan, Features and development of Coot. *Acta Crystallogr. D* **66**, 486–501 (2010). doi:10.1107/S0907444910007493 Medline

63. P. D. Adams, P. V. Afonine, G. Bunkóczi, V. B. Chen, I. W. Davis, N. Echols, J. J. Headd, L.-W. Hung, G. J. Kapral, R. W. Grosse-Kunstleve, A. J. McCoy, N. W. Moriarty, R. Oeffner, R. J. Read, D. C. Richardson, J. S. Richardson, T. C. Terwilliger, P. H. Zwart, PHENIX: A comprehensive Python-based system for macromolecular structure solution. *Acta Crystallogr. D* **66**, 213–221 (2010). doi:10.1107/S0907444909052925 Medline

64. D. H. Barouch, Z. Y. Yang, W. P. Kong, B. Korioth-Schmitz, S. M. Sumida, D. M. Truitt, M. G. Kishko, J. C. Arthur, A. Miura, J. R. Mascola, N. L. Letvin, G. J. Nabel, A human T-cell leukemia virus type 1 regulatory element enhances the immunogenicity of human immunodeficiency virus type 1 DNA vaccines in mice and nonhuman primates. *J. Virol.* **79**, 8828–8834 (2005). doi:10.1128/JVI.79.14.8828-8834.2005 Medline

65. L. Naldini, U. Blömer, F. H. Gage, D. Trono, I. M. Verma, Efficient transfer, integration, and sustained long-term expression of the transgene in adult rat brains injected with a lentiviral vector. *Proc. Natl. Acad. Sci. U.S.A.* **93**, 11382–11388 (1996). doi:10.1073/pnas.93.21.11382 Medline

66. L. Wang, W. Shi, M. G. Joyce, K. Modjarrad, Y. Zhang, K. Leung, C. R. Lees, T. Zhou, H. M. Yassine, M. Kanekiyo, Z. Y. Yang, X. Chen, M. M. Becker, M. Freeman, L. Vogel, J. C. Johnson, G. Olinger, J. P. Todd, U. Bagci, J. Solomon, D. J. Mollura, L. Hensley, P. Jahrling, M. R. Denison, S. S. Rao, K. Subbarao, P. D. Kwong, J. R. Mascola, W.-P. Kong, B. S. Graham, Evaluation of candidate vaccine approaches for MERS-CoV. *Nat. Commun.* **6**, 7712 (2015). doi:10.1038/ncomms8712 Medline

67. E. M. Covés-Datson, J. Dyall, L. E. DeWald, S. R. King, D. Dube, M. Legendre, E. Nelson, K. C. Drews, R. Gross, D. M. Gerhardt, L. Torzewski, E. Postnikova, J. Y. Liang, B. Ban, J. Shetty, L. E. Hensley, P. B. Jahrling, G. G. Olinger Jr., J. M. White, D. M. Markovitz, Inhibition of Ebola virus by a molecularly engineered banana lectin. *PLOS Negl. Trop. Dis.* **13**, e0007595 (2019). doi:10.1371/journal.pntd.0007595 Medline

68. M. Matrosovich, T. Matrosovich, W. Garten, H. D. Klenk, New low-viscosity overlay medium for viral plaque assays. *Virol. J.* **3**, 63 (2006). doi:10.1186/1743-422X-3-63 Medline

69. Y. Cai, J. Zhang, T. Xiao, H. Peng, S. M. Sterling, R. M. Walsh Jr., S. Rawson, S. Rits-Volloch, B. Chen, Distinct conformational states of SARS-CoV-2 spike protein. *Science* **369**, 1586–1592 (2020). doi:10.1126/science.abd4251 Medline
