# Supplementary material for: Broadly neutralizing antibodies target the coronavirus fusion peptide
Source: Science. 2022 Jul 12:eabq3773. doi: 10.1126/science.abq3773 (PMC9348754; doi:10.1126/science.abq3773)
Supplement: Supplementary file 2 — MDAR Reproducibility Checklist [file science.abq3773_mdar_reproducibility_checklist.pdf]

Materials Design Analysis Reporting (MDAR)
Checklist for Authors

The MDAR framework establishes a minimum set of requirements in transparent reporting applicable to studies in the life sciences (see Statement of Task: doi:10.31222/osf.io/9sm4x.). The MDAR checklist is a tool for authors, editors, and others seeking to adopt the MDAR framework for transparent reporting in manuscripts and other outputs. Please refer to the MDAR Elaboration Document for additional context for the MDAR framework.

For all that apply, please note where in the manuscript the required information is provided.

Materials:

Newly created materials	indicate where provided: page no/section/legend)	n/a
The manuscript includes a dedicated "materials availability statement" providing transparent disclosure about availability of newly created materials including details on how materials can be accessed and describing any restrictions on access.	Materials availability statement included after the Acknowledgements section.	

Antibodies	indicate where provided: page no/section/legend)	n/a
For commercial reagents, provide supplier name, catalogue number and RRID, if available.	Catalog numbers provided for commercial antibodies.	

DNA and RNA sequences	indicate where provided: page no/section/legend)	n/a
**Short novel DNA or RNA including primers, probes:** Sequences should be included or deposited in a public repository.	Antibody sequences deposited in GenBank.	

Cell materials	indicate where provided: page no/section/legend	n/a
**Cell lines:** Provide species information, strain. Provide accession number in repository **OR** supplier name, catalog number, clone number, **OR** RRID.	Catalog or accession numbers for cell lines provided in Materials and Methods section.	
**Primary cultures:** Provide species, strain, sex of origin, genetic modification status.	Primary human B cells, non-genetically modified	

Experimental animals	indicate where provided: page no/section/legend)	n/a
**Laboratory animals or Model organisms:** Provide species, strain, sex, age, genetic modification status. Provide accession number in repository **OR** supplier name, catalog number, clone number, **OR** RRID.	Hamster data provided in Materials and Methods.	
**Animal observed in or captured from the field:** Provide species, sex, and age where possible.		N/A

Plants and microbes	indicate where provided: page no/section/legend)	n/a
**Plants:** provide species and strain, ecotype and cultivar where relevant, unique accession number if available, and source (including location for collected wild specimens).		N/A
**Microbes:** provide species and strain, unique accession number if available, and source.		N/A

Human research participants	indicate where provided: page no/section/legend) or state if these demographics were not collected	n/a
If collected and within the bounds of privacy constraints report on age, sex and gender or ethnicity for all study participants.	Described in Materials and Methods section on Study participants.	

Design:

**Study protocol**	**indicate where provided: page no/section/legend)**	**n/a**
If study protocol has been pre-registered, provide DOI. For clinical trials, provide the trial registration number **OR** cite DOI.	Trial registration numbers provided in Materials and Methods section on Study participants.	

**Laboratory protocol**	**indicate where provided: page no/section/legend)**	**n/a**
Provide DOI **OR** other citation details if detailed step-by-step protocols are available.		N/A

**Experimental study design (statistics details)**		
**For in vivo studies:** State whether and how the following have been done	**indicate where provided: page no/section/legend. If it could have been done, but was not, write not done**	**n/a**
Sample size determination	The group sizes are sufficient to determine if the proposed species support virus replication and development of clinical signs. Minimal group sizes of 10 (n=5/cohort) are proposed initially by a NIH statistician. The larger group sizes (n=6/cohort that has been used for all efficacy studies for US Government Countermeasures Acceleration Group) will allow for increased statistical analysis for determination of future experimental needs.	
Randomisation	In Materials and Methods: Animals were randomly assigned to groups to balance as closely as possible between ages (same in this experiment), weight ranges, and sex distribution prior to challenge in consultation with a NIH statistician.	
Blinding	In Materials and Methods: The study remained blinded to all other individuals (except the Study Director team that prepared the antibodies and virus for challenge) until termination of the in-life phase of the study. Comparative Medicine Team remained blinded throughout the course of the in-life portion of the study to prevent bias in clinical scoring. Animal ID's and respective treatments were unblinded once all Core and Pathology data packages were received.	
Inclusion/exclusion criteria	Followed the ARRIVE guidelines (Animal Research: Reporting of In Vivo Experiments). There were no exclusions in animals, experiment units and data points in this experiment. The exact value of n/group was reported in the analysis.	

**Sample definition and in-laboratory replication**	**indicate where provided: page no/section/legend**	**n/a**
State number of times the experiment was replicated in laboratory.	Stated in Statistical analyses section of Materials and Methods.	
Define whether data describe technical or biological replicates.	Stated in Statistical analyses section of Materials and Methods.	

**Ethics**	**indicate where provided: page no/section/legend**	**n/a**
**Studies involving human participants:** State details of authority granting ethics approval (IRB or equivalent committee(s), provide reference number for approval.	Stated in Materials and Methods section on Study participants.	
**Studies involving experimental animals:** State details of authority granting ethics approval (IRB or equivalent committee(s), provide reference number for approval.	Stated in Materials and Methods section on Syrian hamster efficacy studies.	

**Studies involving specimen and field samples:** State if relevant permits obtained, provide details of authority approving study; if none were required, explain why.		N/A

Dual Use Research of Concern (DURC)	indicate where provided: page no/section/legend	n/a
If study is subject to dual use research of concern regulations, state the authority granting approval and reference number for the regulatory approval.		N/A

Analysis:

Attrition	indicate where provided: page no/section/legend	n/a
Describe whether exclusion criteria were preestablished. Report if sample or data points were omitted from analysis. If yes report if this was due to attrition or intentional exclusion and provide justification.		N/A

Statistics	indicate where provided: page no/section/legend	n/a
Describe statistical tests used and justify choice of tests.	Stated in Statistical analyses section of Materials and Methods.	

Data availability	indicate where provided: page no/section/legend	n/a
For newly created and reused datasets, the manuscript includes a data availability statement that provides details for access or notes restrictions on access.	Data availability statement included after the Acknowledgements section.	
If newly created datasets are publicly available, provide accession number in repository **OR** DOI **OR** URL and licensing details where available.	Antibody sequences deposited in GenBank and PDB accession numbers provided.	
If reused data is publicly available provide accession number in repository **OR** DOI **OR** URL, **OR** citation.		N/A

Code availability	indicate where provided: page no/section/legend	n/a
For all newly generated custom computer code/software/mathematical algorithm or re-used code essential for replicating the main findings of the study, the manuscript includes a data availability statement that provides details for access or notes restrictions.		N/A
If newly generated code is publicly available, provide accession number in repository, **OR** DOI **OR** URL and licensing details where available. State any restrictions on code availability or accessibility.		N/A
If reused code is publicly available provide accession number in repository **OR** DOI **OR** URL, **OR** citation.		N/A

Reporting

MDAR framework recommends adoption of discipline-specific guidelines, established and endorsed through community initiatives. Journals have their own policy about requiring specific guidelines and recommendations to complement MDAR.

Adherence to community standards	indicate where provided: page no/section/legend	n/a
State if relevant guidelines (e.g., ICMJE, MIBBI, ARRIVE) have been followed, and whether a checklist (e.g., CONSORT, PRISMA, ARRIVE) is provided with the manuscript.	ARRIVE guidelines were followed for hamster experiments.	
